# Rapid nasal metabolism organizes the neural representation of odors

**DOI:** 10.21203/rs.3.rs-10695342/v1

**Published:** 2026-09-04

**Authors:** Elvis F. Acquah, Madison A. Herrboldt, Joel Hallkaj, Mona A. Marie, Zhenxing Wu, Jeanne Chaloyard, Isabelle Andriot, Jean-Marie Heydel, Kai Zhao, Hiroaki Matsunami, Matt Wachowiak

**Affiliations:** 1 Department of Neurobiology, University of Utah School of Medicine, Salt Lake City, UT, 84112, USA.; 2 Department of Molecular Genetics and Microbiology, Duke University School of Medicine, Durham, NC, 27710, USA; 3 Department of Otolaryngology - Head and Neck Surgery, The Ohio State University, Columbus, Ohio.; 4 Université Bourgogne Europe, CNRS, Laboratoire Interdisciplinaire Carnot de Bourgogne ICB UMR 6303, Dijon, 21000, France; 5 Université Bourgogne Europe, Institut Agro, CNRS, INRAE, UMR CSGA, 21000 Dijon, France; 6 Probe Research Infrastructure, ChemoSens facility, CNRS, INRAE, 21000 Dijon, France

## Abstract

The olfactory system translates chemical stimuli (odorants) into neural patterns of activity that give rise to odor perception. Canonical models of odor coding consider the binding of odorants to receptors expressed on olfactory sensory neurons (OSNs) as the primary organizing step in this process^1^. However, such models fail to account for key aspects of odorant-evoked responses measured in vivo. Here, we considered how a process peripheral to receptor-ligand binding - the metabolism of odorants by xenobiotic enzymes in the nasal mucosa - may impact odor codes in vivo. Using imaging in awake mice and odorants linked by well-characterized enzymatic pathways^2–4^, we find that nasal metabolism rapidly generates odorous metabolites and profoundly alters odor representations over a time-scale relevant for odor perception. Rapid odorant metabolism explains what otherwise appears to be complex tuning of OSNs as well as diverse temporal response patterns not accounted for by a simple receptor-ligand model. While nasal metabolism introduces confusion between identity-based representations of inhaled versus metabolism-generated odorants, we find that inhalation-linked response timing resolves this confusion and that temporally segregated representations of inhaled versus metabolism-generated odorants are preserved at the level of olfactory bulb projections to cortex. These findings demonstrate that odorant metabolism is integral to olfactory sensation and define a novel role for timing in olfaction: to distinguish external chemical signals from those generated internally, potentially supporting distinct percepts and behavioral responses to each.

Olfactory stimuli are encoded by combinatorial patterns of OSN activity that vary in their identity and temporal dynamics^1,5,6^, but how these patterns relate to chemical stimulus features and underlie odor perception remains unclear. In canonical models of odor coding, the binding of odorants to odorant receptors (ORs) is the primary determinant of odor representations by OSNs. Odorant-specific identity codes (i.e., *which* neurons are activated) arise from the specificities of ORs to odorant chemical features^1^. Timing-based codes have been proposed to arise from the varying sensitivity of ORs to a given odorant - which, in mammals, leads to different activation times as nasal odorant concentrations ramp to peak levels following inhalation^7^. However, in vivo studies have revealed features of odorant-evoked response patterns that are not easily explained by a strictly receptor-determined models, including complex chemical tuning for a given OSN^8^, similar response patterns evoked by chemically and perceptually distinct odorants^9^, and temporal responses that do not map to receptor sensitivity^5^.

One step often overlooked in the path from vapor-phase odorant to receptor-ligand binding in vivo is the chemical transformation of odorants by enzymes in the nasal mucosa. These ‘xenobiotic’ enzymes are among the highest-expressed proteins in the nasal epithelium^10^, are secreted into the mucus surrounding OSN cilia^11^, and convert inhaled - and potentially harmful - compounds to forms that are more easily removed from tissue through increased water-solubility or active transport^12–16^. Odorant metabolism has often been considered to have little impact on odor coding and perception, but metabolites of inhaled odorants can themselves function as odorants and there is evidence in humans that odorant metabolism occurs within a single breath and shapes odor perception^14,17,18^. Given the variety of enzymes present in the olfactory mucosa^12^ and the range of their chemical substrates and products, nasal metabolism has the potential to broadly impact odor representations (Fig. 1A).

We investigated this impact in awake mice, focusing on odorants linked by the actions of a few enzymes whose activity in mammalian nasal mucosa is well-established^2,3,12^. We find that odorant metabolism occurs within the timescale of a single respiratory cycle of the mouse and that it is a major organizing feature of odor representations, suggesting that what has typically been considered a ‘housekeeping’ process is in fact integral to olfactory sensation. While nasal metabolism may appear maladaptive for recognizing external stimuli, we find that inhalation-linked timing separates signals from inhaled and metabolism-generated odorants and that temporally distinct representations are preserved among olfactory bulb outputs. Thus, the use of timing as a coding mechanism converts what appears as a ‘bug’ in olfactory system function into a ‘feature’ that allows for distinct representations of - and potentially distinct responses to - external and internally generated olfactory signals.

## Odorant metabolism explains in vivo tuning of class I OR-expressing OSNs.

To assess how nasal odorant metabolism shapes incoming olfactory information, we focused on the conversion of inhaled esters and aldehydes into their carboxylic acid derivatives (Fig. 1B). Metabolism of both classes of odorant by rodent nasal mucosa is well-established and linked to the nasal expression of carboxylesterase (CES) (for esters) and aldehyde dehydrogenase or oxidase (AOX/ALDH) (for aldehydes)^2–4,19,20^. Furthermore, esters, aldehydes and carboxylic acids constitute major classes of odorant that are abundant in the natural environment and heavily used in experimental studies, and a subclass of mammalian ORs (class I) constituting approximately 10% of the mammalian OR gene family appears specialized to detect carboxylic acids^21^.

We first analyzed a publicly-available dataset of in vivo OSN responsiveness^9^ to many odorants including esters, aldehydes and their carboxylic acid metabolites produced by CES or AOX/ALDH, respectively. The dataset was imaged from OSN inputs to dorsal olfactory bulb (OB) glomeruli, which includes the projection domain of class I OR - expressing OSNs^22^. Odorant response maps to a given acid and its metabolic parents were nearly identical (Fig. 1C, **Extended Data Fig. 1A, B**). A pairwise matrix of co-tuning across glomeruli responsive to 7 different acids and various derivatives revealed a systematic tuning rule, with reliable co-tuning to the metabolically related derivatives of each acid (Fig. 1D) but high selectivity against small changes in carbon backbone structure. CES or AOX/ALDH metabolism accounted for the most reliably co-tuned odorant pairs (**Extended Data Fig. 1C**) and explained 72% of the off-diagonal energy in the co-tuning matrix.

Next, building on the recent characterization of a human class I OR (OR51E2), which identified a structural basis for selectivity to carboxylic acids^23^, we expressed a class I OR from mouse, Or52h2, in a heterologous expression system^24^ and challenged it with odorants in vitro. Or52h2 responded sensitively to its preferred acid ligand, 2-pentenoic acid, but was insensitive to the ester, aldehyde or alcohol derivatives (Fig. 1E). To characterize the selectivity of Or52h2 in vivo we generated a mouse line tagging Or52h2-expressing OSNs (Or52h2-IRES-mKate2), crossed this line into a Ca^2+^ reporter strain (OMP-Cre:TIGRE2-GCaMP8m), and performed two-photon imaging from Or52h2 OSN terminals in its cognate glomerulus in awake mice (n = 7). In this setting, Or52h2-expressing OSNs responded robustly to 2-pentenoic acid *and also* its ethyl ester, aldehyde, and alcohol derivatives (Fig. 1F).

These results suggest that odorant metabolism can confer responsiveness of OSNs to inhaled odorants to which their cognate OR is insensitive. We further investigated this expansion of tuning capacity using functionally-defined OSN populations whose cognate glomeruli are robustly identified across individuals and whose odorant sensitivities are well-characterized^9^. We focused initially on four acid-sensitive glomeruli (glomeruli 10, 23, 5, 11) (**Extended Data Fig. 2**). In awake mice expressing GCaMP8m in OSNs, all four glomeruli were co-tuned to their ester and aldehyde derivatives; the two glomeruli tested also responded to the alcohol derivative, as seen for Or52h2 (**Extended Data Fig. 2A**). Responsiveness to the alcohol could reflect a two-step metabolization process, with alcohol dehydrogenase first generating the aldehyde followed by subsequent conversion to the acid.

OSNs have been variously described as narrowly-tuned to specific chemical features or broadly-tuned with complex specificities^1,9,25,26^. Odorant metabolism may underlie complex tuning in vivo: in OSNs that are narrowly-tuned to a carboxylic acid, carboxylesterase metabolism could confer broad responsiveness to structurally distinct esters as long as their hydrolysis product yields the appropriate acid. We tested this prediction in awake mice (n = 6) by challenging the tiglic acid-sensitive identified glomerulus (Fig. 1G) with an array of ester derivatives whose alkyl end spanned a range of structural features; we included several aldehydes metabolized into effective or ineffective carboxylic acids (**Extended Data Fig. 2B**). Esters and aldehydes whose metabolic products matched effective carboxylic acids for the glomerulus were themselves effective odorants, while those predicted to be metabolized into ineffective acids were ineffective (**Extended Data Fig. 2B**). Collectively, the 21 effective odorants covered a large fraction of the ‘space’ of physicochemical odorant features, as defined by a database of 4989 odorous compounds^27^ (Fig. 1G). However, all 17 of the effective esters and aldehydes are metabolized into one of four effective carboxylic acids, which vary only slightly in structure and span a much smaller portion of the space (Fig. 1G). Thus, odorant metabolism contributes substantial power to explaining the tuning of OSNs to physicochemical odorant features.

## Odorant metabolism is rapid and predicts inhalation-linked timing differences in vivo.

Whether nasal metabolism can generate metabolite odorants on a timescale relevant to odor perception - which can occur within a single respiratory cycle, or as little as 100 – 300 milliseconds in rodents^28–31^ - is unclear. To better define the time-scale of metabolism-generated olfactory signals we measured the production of odorant metabolites directly from ex vivo olfactory epithelium using Proton Transfer Reaction - Time-of-Flight Mass Spectrometry (PTR-MS) measurements at a scan interval of 50 msec and a rapid odorant exchange device (Fig. 2A, **Extended Data Fig. 3A**). The setup was optimized to detect the parent ester and its alcohol hydrolysis product; detection of the acid product was not feasible due to its low volatility. PTR-MS sampling revealed production of the expected methanol (MeOH) product for three esters (methyl tiglate, methyl-2-methyl butyrate and methyl propionate) along with a concomitant reduction in the concentration of the parent ester, relative to the no-tissue condition (**Extended Data Fig. 3B, C**). For methyl tiglate and methyl-2-methyl butyrate, which yielded the strongest signals, MeOH onset lagged that of the ester by approximately 300 msec (Fig. 2B, C). Given that this lag includes setup-imposed delays such as desorption of the alcohol from the mucosal explant and bulk flow through the chamber, these results support mouse olfactory epithelium generating appreciable metabolite odorants within the time-scale of a single respiratory cycle.

Next, we simulated the dynamics of odorant metabolite production using a 1-D, 2-compartment, physiologically-based pharmacokinetic (PBPK) model of the mouse nasal mucosa that included enzyme rate constants for ester metabolism experimentally determined in rodent nasal epithelium^3^ and input kinetics that mimicked odorant concentration dynamics during natural breathing^32^ (Fig. 2D). We focused on the model’s predicted dynamics of inhaled and metabolism-generated odorant in the mucus layer, which contains the OSN cilia and is the site of OR-ligand binding. Following a single inhalation of the ester methyl tiglate, concentration dynamics in the mucus followed that of the airborne odorant with a slightly delayed time to peak (Δt = 46 ms) and a slower decay (tau = 194 ms) (Fig. 2E). Increasing odorant concentration over several log units had minimal impact on the kinetics of odorant in the mucus compartment, with a reduction in latency of <50 ms over several log units (**Extended Data Fig. 3D).** Introducing the carboxylesterase enzyme produced a rise in mucosal concentrations of the acid metabolite that was delayed relative to the inhaled ester (Δ latency, 95 ms; Δ peak time, 346 ms) (Fig. 2E). Here, concentration substantially impacted the lag time for the metabolite odorant, reflecting concentration-dependence in the rate of odorant metabolism and the accumulation of metabolite over time (**Extended Data Fig. 3D**). Over repeated inhalations (at 4 Hz), acid concentrations accumulated further before reaching a steady-state (Fig. 2F), with concentration profiles weakly modulated by each inhalation and lagging those of the inhaled odorant by ~40 – 70 ms (lag of cross-correlation between inhalation-triggered average waveforms, 60 ms) (Fig. 2G); lags did not vary with concentration (**Extended Data Fig. 3E**). These simulations, while simplified, nevertheless suggest that nasal metabolism can generate secondary odorants with sufficient speed to activate metabolite-sensitive OSNs within a single respiratory cycle, but with delayed and dampened respiratory patterning in metabolite concentration across subsequent inhalations. They also suggest that the dynamic range of response latencies to inhaled odorants across concentration is limited.

The dynamics of OSN responses to acids and their metabolic parents, measured from the four acid-sensitive identified glomeruli in awake mice, matched the simulation predictions. Response latencies to each acid, measured after aligning OSN-GCaMP8m signals to the first inhalation after odorant onset, ranged from ~75 – 100 ms (**Extended Data Fig. 4A**). Each glomerulus responded to the ester, aldehyde and alcohol derivatives, presented at a comparable threshold-matched concentration (10 – 100x threshold, see Methods), with latencies that were delayed by ~50 – 200 ms (Fig. 2H, I; **Extended Data Fig. 4B**).

To better follow the respiration-linked timing of OSN inputs in the awake mouse we used the genetically-encoded glutamate sensor iGluSnFR (A148S or iGluSnFR3 variants)^33,34^, expressed in mitral and tufted (MT) cells and imaged from their dendritic tufts^35^. MT-iGluSnFR signals mainly reflect OSN-driven glutamate signaling with a minor contribution from external tufted cells,^36^ and can better follow respiration-driven bursts of sensory input at the 4 – 5 Hz breathing frequencies typical of awake, resting mice^35^.

Timing differences between the primary acid and its metabolism-linked derivative were readily apparent in single trials and were consistent with the PBPK simulation, with ester- (and aldehyde-) evoked responses slower in onset and showing weak modulation by successive inhalations (Fig. 2J, **Extended Data Fig. 4C, D**). Inhalation-aligned averages revealed acid-ester latency differences similar in magnitude to those seen for OSN-GCaMP8m signals in the same identified glomeruli (Fig. 2K). Latencies were also longer for responses to subsequent inhalations, as measured from inhalation-triggered average (ITA) waveforms generated from each inhalation after the first 500 ms of odorant presentation (see Methods) (Fig. 2L, M). A modulation index (MI), defined such that MI of 1 reflects responses that return completely to baseline with each inhalation and MI of 0 reflects tonic responses (see Methods), revealed substantially weaker modulation by the ester for all acid-ester pairs (Fig. 2M).

The metabolism simulation predicted that concentration and sensitivity have a small effect on response latencies for inhaled odorants, while they have a larger impact on metabolite-evoked latencies (**Extended Data Fig. 3D**). To test these predictions in vivo we analyzed response timing as a function of odorant concentration, ranging from near-threshold levels activating a single, ‘primary’ glomerulus to 100 – 1000x more concentrated levels recruiting input to multiple ‘non-primary’ glomeruli (example, Fig. 3A–C). Consistent with the simulation, across six identified glomeruli with primary sensitivities to an acid (4 acids) or ketone (2 ketones; 2-hexanone and 2’-OH acetophenone; glomeruli #12 and #20 from^9^; **Extended Data Fig. 5**), onset latencies varied over a limited range, with MT-iGluSnFR response latencies decreasing by ~50 msec as concentrations increased over 2 – 3 log units (Fig. 3D, ‘tiglic acid’; summary data, **Extended Data Fig. 6A**). Timing differences between the most-sensitive and less-sensitive glomeruli were even smaller, with a median delay of 20 ms (IQR: 7 – 47 ms) for MT-iGluSnFR signals (Fig. 3E, ‘onset’) (47 ms, IQR: 13 – 80 ms for OMP-GCaMP8m signals). Even these small differences disappeared during subsequent inhalations (example, Fig. 3C; Fig. 3E, ‘ITA’, **Extended Data Fig. 6B**), suggesting that concentration- or sensitivity-based timing differences are not robust across repeated inhalations of odorant.

In contrast, ester-evoked response latencies, measured from acid-sensitive glomeruli and thus reflecting responses to ester metabolism (example, Fig. 3A, bottom), were consistently longer than acid-evoked latencies in the same glomeruli (Fig. 3F, **Extended Data Fig. 6C**) and varied more widely across concentration (Fig. 3D, **Extended Data Fig. 6D-F**) - also consistent with the metabolism simulation. These longer delays could not be explained by relative sensitivity: in many cases, responses to the highest concentrations of ester were slower than those evoked by the lowest, perithreshold concentrations of the acid (Fig. 3B; **Extended Data Fig. 6D**). Thus, inhalation-linked timing differences distinguishing OSN responses to inhaled odorants from those to odorant metabolites are robust across concentration and across glomeruli with different sensitivities.

## Inhalation-linked timing separates glomerular representations of inhaled versus metabolism-generated odorants.

These results suggest a model of OSN response timing in which activation by inhaled odorants drives short-latency, strongly-modulated responses following each inhalation, while activation by nasal metabolites drives modestly-delayed and poorly-modulated responses. Inhalation of odorants subject to nasal metabolism will generate a response pattern consisting of direct and metabolite-evoked responses. We hypothesized that inhalation-linked timing provides a means to decompose such mixed response patterns.

To test this possibility we separately measured response timing in glomeruli responding selectively to the ester - showing no response to the acid at any concentration, presuming that such responses reflect direct activation of an ester-sensitive OR^37^. Ester-selective glomeruli showed short-latency, strongly-modulated responses that persisted across subsequent inhalations (example, ROIs 4 and 5, Fig. 3B, C) and were not significantly different from those of acid-evoked latencies in acid-sensitive glomeruli, while latencies in acid-ester co-tuned glomeruli were shifted significantly later (Fig 3G).

To extend these observations we surveyed responses over a larger extent of the dorsal OB using widefield imaging in OMP:GCaMP8m mice. Imaging covered an area including nearly all of the projection domain of class I - expressing OSNs and a larger portion of the class II - OSN projection domain^22^, which we functionally defined using high concentrations of a carboxylic acid (butyric acid) or ketone (2-hexanone), as done previously^22^ (Fig. 4A). Ester-selective and acid-ester co-tuned glomeruli were located in largely nonoverlapping spatial clusters within each domain. Mapping onset latency or modulation index (MI) to glomerular location revealed a topography of inhalation-linked timing features reflecting the OR class domains (Fig. 4B, C). Glomeruli in the class II domain responded to esters with short latency and strong modulation, while class I-domain glomeruli responded, in the same trials, with longer latency and little to no modulation (Fig. 4D), indicating that timing features are not solely determined by OSN projection domain or odorant class.

Onset latency and MI values, plotted against one another, formed easily-separable clusters that discriminated the response to an acid from its ester derivative (for co-tuned glomeruli) or identified glomerular class (for ester-responsive glomeruli) (Fig. 4E, **Extended Data Fig. 7A**). We quantified the ability of each metric, measured from an individual glomerulus, to classify an odorant as directly-inhaled or metabolically-generated using receiver-operating characteristic (ROC) analysis. Both latency and MI were each successful at classifying responses of class I, acid-ester co-tuned glomeruli as arising from the acid or its ester derivative (Fig. 4F, **Extended Data Fig. 7B, D**), and also classifying ester-evoked responses as arising from either ester-selective or ester-acid co-tuned glomeruli (reflecting metabolism- or directly-inhaled responses, respectively) (Fig. 4F, **Extended Data Fig. 7C, E**). For some acid-ester pairs the distribution of latency or MI values, taken from the same glomeruli in the same session, was completely nonoverlapping (auROC = 1) (Fig. 4F). Thus, onset latency and modulation by subsequent inhalations independently support classifying responses as arising from inhaled or metabolism-generated odorants.

Notably, inhaled and metabolism-generated responses could not be distinguished on the basis of their relative sensitivity to the ester. Indeed, the most-sensitive glomerulus for all five esters tested was always an acid-ester co-tuned glomerulus within the class I domain, with ester-selective, class II-domain glomeruli recruited at 10 – 100x higher concentrations (Fig. 4G). Despite their lower sensitivity, ester-selective glomeruli responded with shorter latencies than the ‘primary’ glomeruli, inconsistent with receptor sensitivity determining response timing^7^ (Fig. 4G). Instead, this result is consistent with odorant metabolism if class I ORs are more sensitive to their carboxylic acid ligand than are class II ORs to the parent ester. The sensitivity difference need not be large, as the PBPK simulations suggested that acid concentrations arising from ester metabolism can reach ~20% of the peak inhaled ester concentration (Fig. 3E).

## Temporally distinct representations of metabolism-generated odorants persist at the level of olfactory bulb output.

If inhalation-linked timing is used to distinguish inhaled from metabolism-generated odorants, where – and in what form – does this distinction occur? Metabolism-generated signals could be suppressed at an early stage, removing their contribution to odor perception. Alternatively they could remain temporally distinct, allowing for parallel higher-order processing of inputs from inhaled and metabolism-generated odorants. Evidence supporting the suppression hypothesis comes from a recent report that OB circuitry suppresses delayed excitatory inputs to mitral/tufted cell (MT) cells on a time-scale matching that of metabolism-generated delays^38^. To test this hypothesis directly, we interrogated mitral and tufted cells at their glomerular ‘input’ and somatic ‘output’ compartments using GCaMP8f (Fig. 5A), as in^38^. We focused on the same identified glomeruli and diagnostic odorants as for OSN imaging, which evoked MT-GCaMP8f signals in a single glomerulus (Fig. 5Bi), then identified cohorts of ‘daughter’ tufted cell (TC) or mitral cell (MC) somata by adjusting the imaging plane and identifying cells excited by the primary acid at the perithreshold concentration (see Methods) (Fig. 5Bii, iii; **Extended Data Fig. 8A, B**).

As expected, glomerular MT-GCaMP8f signals in acid-sensitive glomeruli showed longer-latency response onsets to the ester - as well as to their respective aldehyde and alcohol derivatives, tested at 10 – 100x perithreshold (**Extended Data Fig. 8C, D, E**) - and responses to subsequent inhalations were delayed such that the acid and ester-evoked ITA waveforms appeared out-of-phase (Fig. 5C, D, E, i). Contrary to the temporal filtering hypothesis, suppressive responses to esters did not emerge in TC or MC somata. Instead, daughter TCs as well as MCs showed delayed excitatory response onsets (Figs. 5C, E, ii, iii) and showed similarly delayed ITA waveforms for responses to successive inhalations (Fig. 5D, ii). Suppressive responses were evident in neighboring glomeruli and in adjacent (i.e., non-daughter) MCs in the same field of view (Fig. 5C, iii, **Extended Data Fig. 9A, B**), indicating that inhibitory circuits were engaged by odorant stimulation. Even in cases where an ester evoked short-latency excitation in neighboring ester-selective glomeruli, reflecting direct activation, responses in a nearby acid - ester co-tuned glomerulus and its daughter cells consisted of delayed excitation rather than suppression (**Extended Data Fig. 9C**).

Ester-evoked response latencies remained longer than their threshold-matched acid responses across up to three log units of concentration (**Extended Data Fig. 10**). Furthermore, latency differences between acid and ester-evoked responses were statistically similar for all three MT levels, and also similar to those of presynaptic inputs (iGluSnFR or OSN-GCaMP8m signals (p = 0.15, one-way ANOVA, F(4,58) = 1.76). Together, these results indicate that sensory signals arising from metabolism-generated odorants are not suppressed by OB circuits but instead are relayed to olfactory cortex in a form that remains temporally distinct from that of inhaled odorants, enabling the independent processing of externally-sampled and internally-generated olfactory information.

## Discussion

The primary function of odorant metabolism by enzymes in the nasal cavity is to facilitate the removal of potentially harmful external molecules from the mucus and underlying epithelial tissue^13^. Our results indicate that, by rapidly generating odorous metabolites, this process profoundly alters odor representations and does so over a time-scale relevant for odor perception. We focused on the actions of a few enzymes that convert esters and aldehydes into carboxylic acids; the impact of these pathways on odor representations was particularly noticeable and tractable to study due to a specialized odorant receptor subfamily with high sensitivity to acids and their OSN projections to a distinct glomerular domain^22,23,39^. We expect that these results represent only a sampling of the impact on odor representations mediated by the full repertoire of enzymes expressed in the nasal epithelium, which is large and impacts odorants with diverse chemistry^10,14^.

The diversity and abundance of enzyme expression in the nasal epithelium suggests that the adaptive value of removing inhaled chemicals from the upper airway outweighs potential confounds to odor identity coding. Nonetheless it seems crucial to disambiguate sensory signals reflecting inhaled odorants from those generated by their metabolites. For example, carboxylic acids are common products of bacterial action and organic decomposition (i.e., spoilage or fermentation)^40,41^ and at least some acids elicit innate avoidance^39,42^, while esters are commonly produced by cellular metabolism in flowers and fruits^43,44^, often signaling a nutritive food source. Thus a carboxylic acid generated by nasal metabolism of an ester has a different meaning than the same acid sampled from the external environment.

We find that inhalation-linked timing provides a robust neural feature by which to disambiguate these signals. Multiple lines of evidence suggest that the earliest-activated inputs to the olfactory system preferentially drive odor perception^28,29,45^; this phenomenon - termed ‘primacy’^7,46^ - has been proposed to mediate concentration-invariant odor recognition^47^ and odor learning^48^. We suggest that perceptual primacy is also adaptive for avoiding confusion between inhaled odorants and their nasal metabolites. In fact, given that timing differences between inhaled and metabolism-generated odorants are larger, more reliable across concentration, and more robust across repeated sampling than are timing differences between glomeruli with different sensitivities to inhaled odorants, disambiguating inhaled from metabolism-generated odorants may be the main adaptive function of a primacy code.

We found that metabolite-driven sensory signals are not filtered by OB circuits, despite a recent report that similarly-delayed inputs to OB glomeruli are suppressed^38^. Instead, timing-based features distinguishing inhaled from metabolism-generated odorants persist at the level of MT cells and are thus relayed to olfactory cortical areas. It is intriguing to consider the fate of these different signals. Metabolite signals could be suppressed at the next stage of processing, in piriform cortex. Piriform circuits mediate suppression of MT inputs that are delayed on the 50 – 100 ms time-scale typical of metabolite-driven MT activation and may also suppress responses to tonic MT activity^49,50^.

Alternatively, temporally-distinct metabolism-generated signals may persist in cortical areas and be processed in parallel to signals from inhaled odorants, potentially mediating distinct but adaptive behavioral responses to such signals. Such a strategy may generalize beyond nasal metabolism to include the differential processing of external versus other internally-generated odorants, including those arising from the oral cavity or exhaled breath, which can carry crucial information about body state and recent food consumption^51,52^.

A third possibility is that signals from odorant metabolism are not fully disambiguated from those of inhaled odorants and instead contribute to odor perception. Several prior studies have reported that odorant metabolism shapes perceived odor qualities in humans^14,18,53^. Previous analyses have found that intracellular metabolic relationships partially explain perceptual similarities between odorant compounds^54,55^; these studies considered metabolic processes within an odor *source* as driving co-occurrence of different compounds. Nasal odorant metabolism, by contrast, generates co-occurrence of odorants within the *receiver* and does so independent of odor source, meaning that co-occurrence of an odorant and its nasal metabolites is extremely reliable. Thus, to the extent that the odorant co-occurrence is an organizing factor driving odor quality perception, metabolic relationships determined by nasal enzymes may be a primary organizing dimension of odor perceptual space that bears further exploration.

## Methods

### Animals

Mice were used from the following genetically-modified strains, obtained from the Jackson Laboratories: OMP-Cre, JAX Stock #006668; TIGRE2-jGCaMP8m-IRES-tTA2-WPRE (‘TIGRE2-GCaMP8m’), Jax# 037718; Tbet-Cre, JAX #024507. The data presented and re-analyzed from^9^, and two of 6 mice used for the data presented in Fig. 2B, were collected in heterozygous crosses of OMP-IRES-tTA (Jackson Laboratory stock #017754)^56^ and tetO-GCaMP6s (Jackson Laboratory stock #024742)^57^ mice aged 2–6 months (OMP-IRES-tTA × tetO-GCaMP6s mice). Or52h2-IRES-mKate2 mice were generated at the University of Utah Gene Targeting Core by inserting an *IRES*-*mKate2* cassette downstream of the CDS of the OR gene using *Easi*-CRISPR. Or52h2-IRES-mKate2 mice are available from the investigators on request. Mouse lines were maintained in a breeding colony at the University of Utah and were crossed as described in the Text. Experiments used male and female mice from 3 – 6 months in age. Mice were housed up to 4/cage and kept on a 12/12 h light/dark cycle with food and water available ad libitum. All procedures were carried out following the National Institutes of Health Guide for the Care and Use of Laboratory Animals and were approved by the University of Utah Institutional Animal Care and Use Committee.

### Viral Vectors

Expression of iGluSnFR variants and GCaMP8f in M/T cells was achieved with Cre-dependent expression of viral vectors in the OB of Tbet-Cre mice. Viral vectors used were: AAV5-Flex-GCaMP8f (pGP-AAV-syn-FLEX-jGCaMP8f-WPRE, Addgene #162379); AAV1-FLEX-iGluSnFR3 (pAAV.hSyn.FLEX.iGluSnFR3.v857.GPI.codonopt, Addgene #17581); AAV5-FLEX-SF-iGluSnFRA184S (pAAV.hSyn.FLEX.SF-iGluSnFR.A184S, ViGene). Virus injection (300 – 500 nL, ~250 μm depth, 1:2 – 1:10 dilution) was done using pressure injections and beveled glass pipettes, as described previously^58^, during the chronic window surgery.

### Awake, head-fixed imaging

For two-photon imaging in awake, head-fixed mice we prepared a headbar and chronic imaging window implanted over both OBs under isoflurane anesthesia, as described in^38^. The window consisted of a 3 mm diameter coverslip that spanned the midline and included a field of view of approximately the anterior and medial half of each OB. Animals were allowed two weeks to recover from surgery and allow for window clearing and virus expression. Animals were then acclimated to head-fixation on a circular treadmill^59^ over the course of 4 – 6 days, and subject to at least one 30-minute session including odorant presentation prior to data collection. Imaging sessions lasted from 30–60 minutes per day, over a span of 5 – 30 days.

For widefield imaging in awake, head-fixed mice, a headbar was implanted and the skull overlying the OB was exposed, cleaned with hydrogen peroxide (1%) and 70% ETOH and sealed with Kwik-Sil (WPI). After recovery from surgery mice were acclimated to head-fixation and odorant presentation. After acclimation, mice were anesthetized with isoflurane, the Kwik-Sil removed and the bone overlying nearly the full extent of each dorsal OB thinned to transparency using a dental drill (Osada USA) at low speed. A thin layer of cyanoacrylate was applied to maintain transparency and both OBs were covered with a single 4 mm coverslip sealed in place with optical adhesive (NOA 61, Norland Products, Cranbury, NJ). Data collection in the awake mouse began the following day, and continued for 3 – 5 days or until the underlying bone regained opacity.

### Data collection

In most experiments two-photon imaging was carried out with a Neurolabware in vivo microscope two-photon microscope (Sutter Instruments or Neurolabware) coupled to a Chameleon Ultra pulsed Ti:Sapphire laser (Coherent) at 940 nm, controlled by Scanbox (Neurolabware) software. Imaging frames were acquired with bidirectional resonance scanning at 30 Hz. For two-photon imaging of GCaMP8m in Or52h2-IRES-mKate2 mice, a dual-color two-photon system was used, consisting of a Sutter Instruments scanning microscope, a Mai Tai HP Ti:Sapphire laser (Spectra-Physics) at 920 nm for GCaMP imaging and a Fidelity-2 fixed-wavelength (1060 nm) laser for mKate2 visualization, with images collected using unidirectional resonance scanning at 15.2 or 15.5 Hz under the control of ScanImage (Vidrio).

For widefield imaging, epifluorescence was collected through a 4x, 0.28 N.A. air objective (Olympus) at 256×256-pixel resolution and 50-Hz frame rate using a back-illuminated CCD camera (NeuroCCD-SM256; RedShirt Imaging) under the control of SM-Turbo software. To minimize hemodynamic artifacts that were prominent in the awake mouse, a dual-wavelength LED illumination system was designed to allow collection of fluorescence emission at the isosbestic excitation point for hemoglobin on alternate frames. Custom Arduino code (kind gift of D. Storace, Florida State Univ) used the camera frame clock to trigger a 470 nm LED or 405 nm LED (M405L4, M470L3, Thorlabs) on alternate frames. LED light was collimated using an aspheric condenser lens (ACL2520U, Thorlabs) and merged into the same illumination path within a 1”-diameter cube (DFM1, Thorlabs) containing a dichroic mirror (DMSP425R, Thorlabs) then passed through a multi-band bandpass filter (69000x, Chroma Technology) before transmission to the objective. Fluorescence emission was collected through the standard Olympus fluorescence turret using a GFP4050a filter cube (Semrock) with emission filter removed. The intensity of each LED was adjusted manually to achieve roughly equal fluorescence emission before data collection. Strobed frames were collected and digitized in sequence. Subsequently, 470 nm : 405 nm ratio images were computed from successive frame pairs on a pixel-by-pixel basis. Odorant-evoked fluorescence changes are thus expressed as change in ratio (ΔR).

Odorants were presented as square pulses lasting 4 – 8 seconds and were typically repeated at the same concentration over 6 – 8 trials, with imaging data collected continuously throughout the series of repeats. An interstimulus interval between 15 – 30 sec was chosen to avoid adaptation between presentations, depending on odorant identity and concentration. Respiratory signals were acquired via tubing connected to a flow sensor (AWM3150V, Honeywell) positioned at the external nares on the side opposite that being imaged, digitized at 1 kHz and synchronized with the imaging frame clock.

### Odorant preparation and presentation

Odorants used are listed in Extended Data Table 1. In most experiments odorants were presented using a custom olfactometer (a.k.a, ‘Turbulator’) that allowed for precise temporal control of odorant onset and offset (**Extended Data Fig. 11**). The device used a novel design that achieved rapid and high dilution of odorant vapor in free space and generated reliable odorant pulses with a 90% rise-time of ~50 ms, with a minimal (<0.2%) change in net air flow to the mouse (**Extended Data Fig. 11**). The design also enabled linear control of dilution levels over a four-fold range and choice of eight odorant channels with no common flow path, allowing for rapid switching between odorant channels and minimal chance for cross-contamination between channels. Fast odorant mixing and dilution occurred via an inexpensive, commercially-available Venturi-based fluid mixing device (BEX mini tank mixing eductor, 0.059” orifice, item# 62386, Unites States Plastic Corp.). Saturated vapor from glass odorant reservoirs with Teflon inflow and outflow ports (Diba Omnifit Q Series GL45 cap, Cole-Parmer) was directed to dedicated T-connectors (Clippard 1/16” swivel tee, ST0–2002) arrayed around the eductor inlets and released into the eductor by closing a normally-open valve attached to vacuum. The small dead volume of the T-connector allowed for rapid odorant onset and offset that was shorter than the typical inter-inhalation interval in an awake, resting mouse (**Extended Data Fig. 11**). Speed and precision of timing was characterized with a photoionization detector (mini-PID, Aurora Scientific). Amount of dilution achieved by the eductor relative to saturated vapor was 0.2%, measured at the position of the mouse nose (5 cm from the eductor nozzle) under standard operating parameters (legend, **Extended Data Fig. 11**). Vapor-phase dilution ranged from two-fold less to two-fold higher depending on flow rate through the odorant reservoir (**Extended Data Fig. 11B**). For the characterization of glomerular specificity across ester and aldehyde derivatives (Fig. 1G, **Extended Data Fig. 2B**), odorants were presented using a high-throughput odor delivery device (aka, ‘Odor Gun’), described previously^60^, which used the same eductor-based air dilution principle and achieved a vapor-phase dilution of ~1.3%. Additional dilutions (by factor of ten, typically, 1E-2 to 1E-6) were achieved by diluting pure odorant in an odorless solvent (caprylic/capric medium-chain triglyceride oil (MCT), C3465, Spectrum Chemical Mfg. Corp.). Odorant concentrations are reported in the Text and Figures as total dilution, accounting for liquid dilution and subsequent air-phase dilution. This reporting is based on ideal (linear) assumptions of vapor pressure after dilution in liquid (i.e., Raoult’s Law) and the subsequent air-phase dilution by the eductor. While many odorants deviate from ideal behavior as described by their Henry’s Law coefficient^61,62^ (which is uncharacterized for the MCT oil solvent), this deviation predominates at more-concentrated liquid dilutions, with subsequent dilutions in solvent increasingly approaching linearity. Thus, relative changes in odorant concentration can be assumed to accurately track liquid dilutions at the high degrees of dilution used in most experiments. Estimated delivered molar concentrations, calculated from the odorant’s vapor pressure and ideal assumptions, are given for reference dilutions in Table S1 and are included to highlight differences in concentration among different odorants and facilitate comparison with other studies.

### In vitro OR - ligand screening

GloSensor cAMP assays (Promega) were used to measure intracellular cAMP levels in Hana3A cells downstream of odorant receptor activation, as previously described^63,64^. Hana3A cells were cultured in minimum essential medium (MEM; Corning) supplemented with 10% fetal bovine serum (FBS; Gibco), penicillin-streptomycin, and amphotericin B^65^. Cells were seeded the day before transfection at a density of 8 × 10^6^–1 × 10^7^ cells/mL. For each 96-well plate, 1,000 ng pGloSensor-20F plasmid (Promega), 500 ng RTP1S plasmid, and 7,500 ng rho-tagged odorant receptor in the pCI mammalian expression vector (Promega) were co-transfected using 20 μL Lipofectamine 2000 (Invitrogen) in MEM supplemented with 10% FBS. After 18–24 h, transfection media were replaced with 25 μL of 2.6% GloSensor substrate (Promega) diluted in HEPES-buffered solution supplemented with D-glucose, and plates were incubated at room temperature in the dark for 2 h prior to measurement.

For in vitro odorant stimulation, 1 M stock solutions were prepared in ethanol and diluted on the day of the assay into Hank’s Balanced Salt Solution (HBSS) supplemented with D-glucose to achieve the indicated final concentrations. A separate saturated stock solution was prepared as the highest starting concentration. Half-log serial dilutions were then generated in HBSS with D-glucose to produce seven odor concentrations and a no-odor control, as previously described^24,63,64^.

Luminescence was measured for 15 min immediately following odorant stimulation using a CLARIOstar^Plus^ plate reader (BMG LABTECH). cAMP accumulation was quantified by integrating luminescence values across measurement cycles (expressed as area under the curve, ‘AUC’), as previously described^64^. Responses were normalized to empty vector controls and baseline luminescence (measured prior to odor addition). Dose–response curves were fitted using a three-parameter logistic regression model in Prism 10.6.1 (GraphPad Software).

### Analysis of chemical feature space

To assess odorant relationships based on physicochemical features and xenobiotic metabolism pathways, we used the Python package RDKit (https://www.rdkit.org/). To visualize and quantify odorant similarities (e.g., Fig. 2C, D), an odorant physicochemical feature space was constructed from 4989 odorous compounds in the combined GoodScents/Leffingwell dataset^27^, as provided by the OpenPOM github site (https://github.com/ARY2260/openpom). This list was supplemented with 12 additional compounds from our experimental odorant panel. RDKit was used to generate molecular fingerprints for each compound from its SMILES string; fingerprints were encoded as MACCS keys, a binary representation indicating the presence or absence of 166 chemical features^66^. Principal component analysis (PCA) embeddings were computed from MACCS fingerprints of the chemical space dataset. For display (Fig. 2C), a three-dimensional scatter projection of the first three PCs was visualized using OriginLab (Origin(Pro) 2026, OriginLab Corporation, Northampton, MA, USA); for visualization purposes, 3615 odorants were displayed instead of the full set.

To quantify physicochemical diversity among odorant groups (Fig. 1G), dispersion within a group was defined as the mean of all pairwise Euclidean distances between individual odorants of their respective group, calculated in the first four PCs, which included 39% of total physicochemical variance.

To visualize the potential for confusion between odorants and their metabolites (Fig. 1A), metabolic reaction products across odorant space were computed based on known reactions of four xenobiotic enzymes: carboxylesterase, aldehyde oxidase, alcohol dehydrogenase and aldo-keto reductase. These enzymes were selected from a larger set detected in rodent olfactory epithelium^12,67^ due to their straightforward chemical reactions with particular chemical substrates. Substrate odorants and their predicted reaction products were identified using RDKit’s rdChemReaction module. For each enzyme, a general reaction pattern was defined using SMARTS notation (Daylight Chemical Information Systems Inc; https://www.daylight.com/dayhtml/doc/theory/theory.smarts.html), which specifies the structural changes a molecule can undergo. These reactions were then applied to a compilation of 632 odorants taken from previous experimental studies^68^. In total, 341 unique reactants were predicted to yield 354 unique products. When reactants and products were combined and duplicates removed, the final set, consisting of 660 unique odorants, was used to construct a circular network graph (907 nodes) using the python package ‘NetworkX’^69^, with lines connecting enzyme target and product odorant nodes.

### Real-time measurement of odorant metabolism by olfactory mucosa

The speed of ester metabolism by mouse olfactory mucosa was measured directly from excised tissue using proton transfer reaction - time of flight - mass spectrometry (PTR-TofF-MS). Esters used were methyl tiglate (CAS# 41725-90-0), methyl 2-methylbutyrate (CAS# 868-57-5), methyl propionate (CAS# 554-12-1) and isopropyl tiglate (CAS# 1733-25-1). We measured production of the alcohol hydrolysis product (methanol, CAS# 67-56-1; isopropanol, CAS# 67-63-0), as carboxylic acid detection was not sufficiently sensitive with the given PTR-MS configuration. All chemicals were purchased from Sigma Aldrich (Sigma Aldrich, St Quentin Fallavier, France).

Experiments were performed on 3 – 6 months male c57bl6 mice bred and housed in the animal facility of the Plateforme de Zootechnie at University of Dijon, France. Mice were housed in ventilated rack cages, with enrichment cell sizzle (SAFE, Augy 89) fed ad libitum with standard food from SAFE (A03–10 pour breeding, next A04–10). After decapitation, olfactory epithelium (OE) was carefully dissected to avoid contamination with respiratory epithelium and immediately frozen (cryotubes in liquid nitrogen) until ready for use. We used a total of 15 mice; 5 mice were used per molecule and per experiment. All experimental protocols were conducted in accordance with ethical rules enforced by French law, and were approved by the local Ethical Committee of the University of Burgundy (Comité d’Ethique de l′Expérimentation Animale Grand Campus Dijon; C2EA grand campus Dijon N°105), and by the French Ministère de l′Education Nationale, de l′Enseignement Supérieur et de la Recherche under the no. 3504.

Measurements were conducted with a proton transfer reaction-mass spectrometer instrument equipped with a Time-of-Flight analyzer (PTR-ToF 8000, Ionicon Analytik, Innsbruck, Austria). Data were recorded with the TOF-DAQ software in the range m/z 0 to 227.42 at one spectrum per 50 ms or one spectrum per 500 ms. The set up was identical to that previously validated and published^70^. During the acquisition we focused on specific masses: m/z = 115.075 (protonated methyl tiglate), m/z = 116.079 (protonated isotope 13C methyl tiglate), m/z = 117.091 (protonated methyl-2-methyl butyrate), m/z = 118.085 (protonated isotope 13C methyl-2-methyl butyrate), m/z = 89.059 (protonated methyl propionate), m/z = 90.063 (protonated isotope 13C methyl propionate), m/z = 143.107 (protonated isopropyl tiglate), m/z = 144.110 (protonated isopropyl tiglate) and for the metabolites produced, m/z = 33.033 (protonated methanol) and m/z = 61.065 (protonated isopropanol). All measurements were carried out at a drift-tube pressure of 2.3 mbar, a drift-tube temperature of 80 °C and a drift-tube voltage of 390 V giving an electric field strength to number density ratio E/N of 116 Td (1 Td = 10^−^17 cm^2^.V) and H_3_O^+^ as reagent ion. The data were corrected for transmission and expressed in concentration (ppb) using primary ions H_3_O^+^ and (H_2_O)_2_H^+^ to account for primary ions fluctuations.

Odorant was drawn over excised olfactory mucosa using a flow control setup as described previously^70^. Briefly, the flow configuration to the PTR-MS consisted of two independent, oven-thermostated circuits (control and experimental) (30 °C) controlled via a 6-way valve, allowing alternate delivery of odorants to either circuit (**Extended Data Fig. 2A**). Each circuit contained a glass trap: one received OE tissue from a single mouse and the other served as a control. Airflow was distributed via stainless-steel strainers and delivered to the PTR-MS through heated PEEK capillaries at 160 mL/min. Humidified zero-air from a Tedlar^®^ gas bag maintained constant moisture, while a second bag contained aqueous odorant solutions (10 ppm in liquid). During experiments, background signals were first recorded in the control circuit, followed by timed introduction of odorants to both control and experimental circuits in a stepwise manner. Between runs, traps and tubing were cleaned to prevent odorant carryover. After the experimental run, the olfactory mucosa explant was heated at 80 °C for 15 minutes in a glass vial to inactivate enzymatic proteins, allowed to cool for 10 minutes, and then reintroduced into the system for analysis as a control for enzyme-dependent biotransformation.

The temporal delay (ΔT) between the PTR-MS signal of a parent odorant compound (A) and that of its enzymatically generated metabolite (B) was estimated using a relative rise-threshold approach combined with linear interpolation. This procedure was developed to provide sub-cycle temporal resolution beyond the nominal 50 ms PTR-MS acquisition interval while minimizing the influence of baseline fluctuations and transient signal noise, following principle commonly used in threshold-based signal onset detection^71^. For each signal trace, the baseline was defined as the mean of the first ten data points within the analyzed response window, and the maximum as the highest intensity observed within the same window. A detection threshold was then calculated independently for each signal as the baseline plus 20% of the signal amplitude (maximum − baseline).

The onset time of the parent compound (tA) was defined as the first crossing of the corresponding threshold during the rising phase of the signal. Because metabolite formation is expected to follow parent compound detection, the metabolite onset time (tB) was defined as the first threshold crossing occurring after tA. To reduce false-positive detections caused by noise or baseline fluctuations, threshold crossings were accepted only when they corresponded to a sustained rise of the signal rather than to an isolated fluctuation around the threshold. Threshold-crossing times were refined by linear interpolation between the last point below threshold and the first point above threshold. The minimum observable temporal delay was then calculated as Δt = tB − tA and reported in milliseconds. As Δt depends on a predefined relative threshold and on the temporal resolution of the acquisition system, it should be regarded as an operational estimate of the minimum observable delay between parent and metabolite appearance rather than as an absolute measure of the underlying enzymatic reaction kinetics.

Maximum methanol concentration produced by OE exposure to methyl tiglate was estimated by PTR-ToF-MS analysis from a calibration curve (y=169.44x, R^2^= 0.9814) obtained by the dilution of a 24.4 mg/l stock solution of methanol.

### Epithelial metabolism model

To simulate the dynamics of odorant and metabolite concentration changes during inhalation, an odorant transient derived from computational fluid dynamics (CFD) modeling of airflow in the rat nasal cavity was coupled to a physiologically-based pharmacokinetic (PBPK) model of transport and metabolism in the mucus and epithelium. The odorant transient was taken from a previous CFD model of the rat nasal passage^72–74^, simulating concentration-change dynamics from a single inhalation at the at the anterior dorsal olfactory region. To model dynamics across successive inhalations the transient was repeated at 4 Hz. Odorant uptake at the air–mucus interface was handled through an effective boundary condition based on odorant solubility, using logP as a convenient estimator^72,75^.

Following uptake, a one-dimensional, two-layer model was used to compute odorant transport and metabolism in the nasal lining. The domain includes a 30 μm mucus layer and a 70 μm epithelial layer. Diffusion of the parent compound (e.g., methyl tiglate) and its metabolite (e.g., tiglic acid) was modeled in each layer. Different diffusivities were assigned to the mucus and epithelial layers. Diffusivity in the mucus was estimated separately for the parent compound and its metabolite (*D*_1_ and *D*_2_, respectively) based on the Wilke–Chang method^76^, while diffusivities in the epithelium were reduced by a factor of 50 to reflect hindered transport in tissue.

Metabolism of the parent compound was described using Michaelis–Menten kinetics^3,77^. The parameters *V*_*max*_ (maximum reaction rate) and *K*_*m*_ (Michaelis constant) were taken from experimentally-determined values for carboxylesterase hydrolysis of vinyl acetate in rat nasal mucosa^3^. The model accounts for both consumption of the parent compound and generation of its metabolite.

The governing equations are given in Eq. (1).

$$\frac{\partial C_{1}}{\partial t}=D_{1}\frac{\partial^{2}C_{1}}{\partial x^{2}}-\frac{V_{max}\cdot C_{1}}{K_{m}+C_{1}}$$

$$\frac{\partial C_{2}}{\partial t}=D_{2}\frac{\partial^{2}C_{2}}{\partial x^{2}}+\frac{V_{max}\cdot C_{1}}{K_{m}+C_{1}}$$

Where *C*_1_, *C*_2_ represent the concentration of the compound and its metabolite, respectively.

The model was implemented in MATLAB (MathWorks, Inc., Natick, MA) using the built-in solver *pdepe*. The spatial domain was discretized across both layers, and time-dependent simulations were driven by the odorant concentration profile obtained from the CFD model as the inlet boundary condition. This input was applied as a time-varying concentration using interpolation, while a zero-concentration condition was specified at the distal boundary. Layer-specific diffusivities and reaction terms were defined as piecewise functions of spatial location. The governing equations were solved simultaneously for the parent compound and its metabolite, accounting for diffusion and Michaelis–Menten kinetics.

The model used the following parameter values: Vmax, 3.7e-4 (mol/L/sec); Km, 0.33e-3 (mol/L); logP (methyl tiglate), 1.69; logP (tiglic acid), 1.4; D1, 7.8e-10 and D2, 8.6e-10 (m^2^/sec) in the mucus, with peak odorant concentration values in the air-phase as given in the Text.

### Imaging Data Analysis

#### Initial data processing pipeline.

Data were initially processed using a custom GUI-based software written in Matlab (https://github.com/WachowiakLab/ImageAnalysisSoftware), as described previously^35^. For time-series analysis of two-photon data, regions-of-interest (ROIs) were chosen manually from the mean fluorescence image and further refined based on odorant response ΔF maps, then all pixels averaged within an ROI and time-series upsampled to 150 Hz (pchip function, Matlab) for further analysis. Respiration signals were low-pass filtered at ~12 Hz and the times of each inhalation start and peak automatically identified using signal processing algorithms described previously^35^. For display in the figures, odorant response maps were generated using the change in fluorescence (ΔF) relative to the average of the 4 seconds preceding odorant onset values, after averaging all repeated odorant presentations. For display only, ΔF rather than ΔF/F values were used to minimize noise from nonfluorescent regions. Response maps were scaled as indicated in the figure and were kept to their original resolution (512 × 512 pixels) and smoothed using a Gaussian kernel with σ of 1 pixel.

#### Analysis of inhalation-linked activity dynamics.

Inhalation-linked response timing features were measured from fluorescence traces of glomerular ROIs after aligning ROI traces to the time of inhalation, measured from the respiration signal. For initial onset latencies, traces from individual odorant presentations (3 – 6 successive trials) were aligned to the first inhalation after odorant onset, then averaged across trials. Because odorant presentation was not triggered by the respiration signal, trials in which odorant onset occurred within 50 ms of inhalation start were excluded from averaging, as were trials in which onset occurred during occasional bouts of high-frequency sniffing. After averaging, ROI traces were z-scored relative to the pre-odor baseline (1 – 2 seconds preceding odorant onset). Onset latency was defined as the time for the trace to reach a threshold level after which it must remain above threshold for 10 consecutive points (67 ms). A threshold of z = 3 was used for two-photon data; for widefield data the threshold was set to z = 4 – 6 due to the higher signal-to-noise ratio of responses. Signals failing to cross threshold within 1 sec of odorant onset were considered non-responsive.

For successive-inhalation analysis, inhalation-triggered average waveforms were constructed by averaging 400 ms-duration snippets of the ROI trace taken from the start of each inhalation occurring after the first 500 ms of odorant presentation, and analyzed as described in^35^. To avoid timing artifacts from preceding or later inhalations, only inhalations occurring at a frequency below 5 Hz (200 ms inter-inhalation interval) were included for averaging. Modulation Index (MI) was calculated from the ITA waveforms. For analysis of two-photon imaging data, MI was defined as the peak-to-trough amplitude of the ITA waveform divided by its mean ΔF relative to pre-odor baseline, then divided by 2. With this metric, an MI of 1 reflects responses that are maximally modulated - i.e., return completely to baseline after each inhalation, while an MI of 0 reflects no inhalation-linked modulation. For widefield OMP-GCaMP8m imaging data (Fig. 4), because of weak but widespread inhalation-linked modulation prior to odorant presentation, MI was defined as the standard deviation of the high-pass filtered ROI trace after the first second of odorant presentation, divided by that of the same trace during the pre-odor period. A high-pass filter cutoff of 1 Hz was used to eliminate tonic signal components. In this case, an MI index of 1 indicates no additional odorant-evoked modulation. MI values were computed on a trial-by-trial basis and averaged across repeated trials of odorant.

#### Mitral and tufted cell somatic signal analysis.

To identify odorant-responsive mitral cells, imaging sequences were first movement-corrected in the XY plane using rigid registration, followed by somatic segmentation in Suite2P^78^. Initial segmentation was manually curated in the Suite2P environment, and the resulting ROIs were exported for further analysis in our custom Matlab Image Analysis GUIs. Daughter mitral cells innervating the parent glomerulus of interest were identified by their responsiveness to a near-threshold concentration of the diagnostic odorant, using an auROC (area under the receiver operating characteristic curve) criterion comparing the fluorescence signal during and prior to odorant presentation, compiled across 4 – 6 trials. In most cases, an auROC cutoff of 0.8 was used to identify responsive cells. For tufted cell analysis, automated segmentation was not used due to confounding signal from extensive lateral dendrites in the same focal plane. Instead, responsive cells were manually segmented based on ΔF response maps, and daughter cells identified by visually tracing their primary dendrites to the parent glomerulus. Following cell identification, for most analyses signals from all identified daughter mitral or tufted cells (range, 2 – 7 per glomerulus) were averaged for analysis of inhalation-linked dynamics, to improve signal-to-noise ratio.

#### Paired acid-ester comparisons.

In most cases, comparison of response metrics between acids and their ester or aldehyde derivatives were made for odorant dilutions matched relative to their near-threshold concentration, and typically at 10x near-threshold unless specified otherwise in the Text. These values were consistent in different mice for the same odorants, and matched dilutions elicited responses of similar peak amplitude (± 30%).

### Statistical analyses

To compare response latencies between acids and their corresponding functional group derivatives (esters, aldehydes, alcohols), separate linear mixed-effects models were fit in R using the lme4 package^79^ for each functional group comparison. Response latency was modeled as a function of functional group (acid versus derivative) and odorant family (tiglic, 2-methyl butyric, phenylacetic, isovaleric) as fixed effects, with biological replicate modeled as a random effect nested within odorant family to account for repeated measurements obtained from the same identified glomerulus across functional groups.


Model:Δlatency~FunctionalGroup+OdorFamily+(1∣OdorFamily:Replicate)

Estimated marginal means (emms) were calculated using the ‘emmeans’ package in R (https://rvlenth.github.io/emmeans/) and pairwise comparisons between acids and the corresponding functional group were performed for each model. For analyses involving multiple planned functional-group comparisons, P values were adjusted using the Bonferroni correction to control the family-wise error rate. For analyses involving a single planned comparison (e.g., acid versus ester), pairwise comparisons of estimated marginal means were performed without adjustment for multiple testing. To directly compare acid and ester response latencies, taking into account different odorants and biological replicates, we fit an analogous mixed-effects model and made pairwise comparisons of estimated marginal means without adjustment for multiple testing.

To compare relative response latencies in non-primary glomeruli for different odorants, latencies relative to the primary glomerulus, measured in the same trials, were analyzed using a one-way analysis of variance (ANOVA) implemented in R, with odorant identity as the independent variable. Odorant-specific emms (calculated using ‘emmeans’) were tested against a null value of zero to determine whether response latencies differed significantly from baseline, and Tukey-adjusted pairwise comparisons were used for post-hoc assessment of differences in latency between odorants. All comparisons were two-tailed.

To evaluate timing-based discrimination of ester and acid-evoked responses, receiver operating characteristic (ROC) analysis was conducted in Python using the SciPy package. True labels were shuffled 5,000 times and the resulting ROC curves for MI and latency were pooled to create a combined null distribution by plotting the 2.5–97.5 percentiles.

Model:Latency~1+(1|Odor)

Post hoc comparisons against a null value of 0.5 (random chance) were implemented using emmeans.

## Supplementary Material

1

Supplementary Material

Supplementary Files

This is a list of supplementary files associated with this preprint. Click to download.

- nrreportingsummaryfinalaug13.pdf

## Figures and Tables

**Figure 1.**
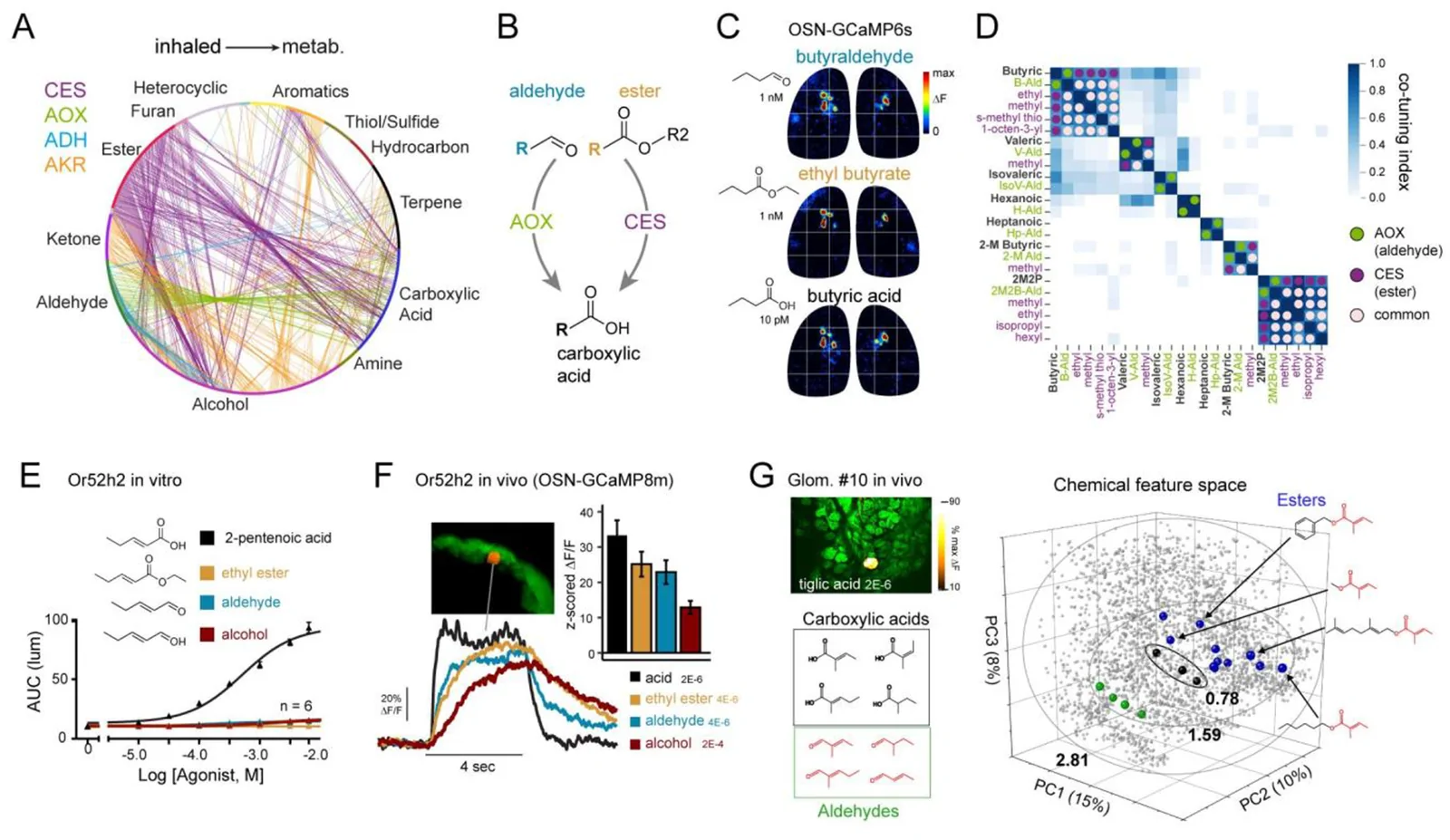
Complex tuning of OSNs in vivo is explained by odorant metabolism. **A.** Network of odorants and their metabolites, computed for four nasal metabolic enzymes on 632 odorants used in a survey of prior experimental studies^68^. CES, carboxylesterase; AOX, aldehyde oxidase; ADH, alcohol dehydrogenase; AKR, aldo-keto reductase. See Main text for details. **B.** Enzymatic conversion of esters and aldehydes by carboxylesterase (CES) and aldehyde oxidase (AOX) or aldehyde dehydrogenase (ALDH), respectively, each leading to production of a carboxylic acid derivative. C. Epifluorescence response maps of OSN inputs (OMP-IRES-tTA × tetO-GCaMP6s) showing near-identical response patterns to butyric acid and its metabolically-linked aldehyde and ester derivatives (butyraldehyde, ethyl butyrate). Estimated delivered concentrations are shown below each structure. Grid shown for reference. Data from^9^. **D.** Matrix of odorant co-tuning for glomeruli analyzed in^9^, compared with odorant pairs connected by CES or AOX/ALDH metabolism (dots). Co-tuning metric indicates mean probability of a glomerulus responding to each odorant pair, as described in^9^. Dots indicate odorant-metabolite pairs, colored according to enzyme (‘AOX’, ‘CES’) or odorant pairs generating the same metabolite (‘common’). **E.** Selectivity of the mouse class I odorant receptor Or52h2 to the carboxylic acid 2-pentenoic acid but not its ester, aldehyde or alcohol derivatives in a heterologous expression system. Concentration-response plot for each odorant; Units expressed as area under the luminescence curve, AUC (see Methods). ethyl ester: E-ethyl-pent-2-enoate; aldehyde: trans-2-pentenal; alcohol: trans-2-penten-1-ol. Error bars indicate SEM (n = 3 experiments per odorant, 3 technical replicates per experiment). **F.** Imaging OSNs expressing Or52h2 in vivo (tagged with mKate2 for visualization) reveals co-tuning to the ester, aldehyde and alcohol derivatives in the awake mouse. Insets, sample OB section showing Or52h2-tagged glomerulus (red) with native GCaMP fluorescence in OSNs (green). Traces are average of 4 presentations per odorant; same odorants as in (D). Inset: Mean response amplitudes to each odorant. Error bars, SEM; n = 7 mice. **G.** Scatter plot of 21 odorants effective at activating the tiglic acid-identified glomerulus (ΔF response maps shown in inset), in the chemical feature space defined by 4989 compounds (3637 shown, gray dots) taken from^27^. Axes are principal components of MACCS chemical fingerprint features (PCs 1 – 3; 33% variance explained). Effective odorants are color coded by odorant class (Acid, Ester or Aldehyde). Structures of effective acids, aldehydes and select esters are shown. Ellipses indicate approximate spread of odorants in the group of all odorants; all effective odorants, and all effective acids and metabolites. Numbers indicate dispersion of odorants within each group, measured in PCs 1 – 4 of the feature space (see Methods). Note that several compounds are stereoisomers (e.g., tiglic acid, angelic acid) and so occupy the same point in physicochemical space.

**Figure 2.**
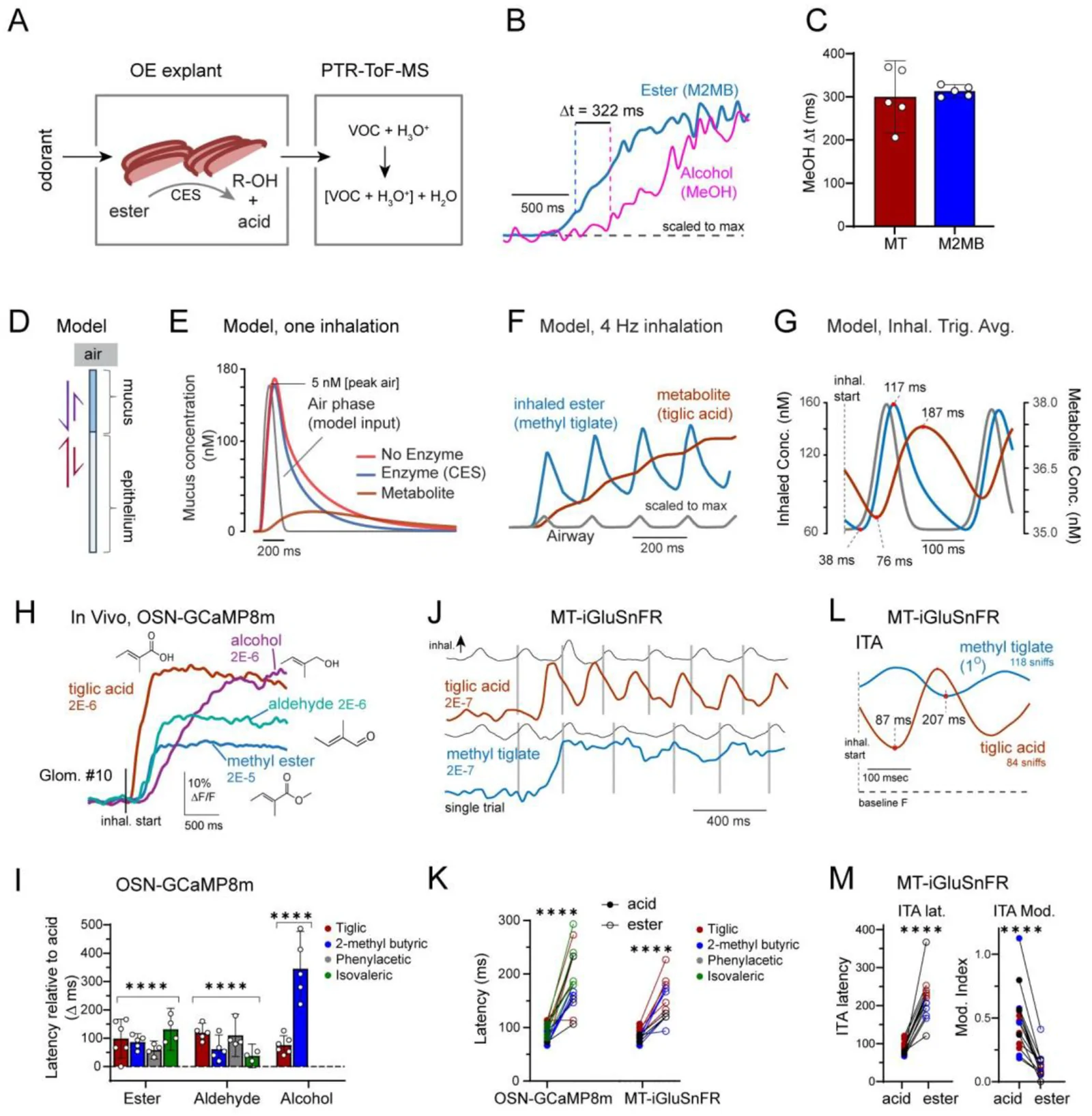
Rapid nasal metabolism explains inhalation-linked temporal response features of OSNs in the awake mouse. **A.** Schematic of setup for direct measurement of ester metabolism by excised mouse nasal olfactory mucosa using PTR-MS. (VOC, Volatile Organic Compound). **B.** Time-course of PTR-MS signal onset for the ester methyl-2-methyl butyrate (M2MB) and its methanol (MeOH) hydrolysis product, scanned in the same trials. Each trace is scaled to its own maximum. Dashed lines indicate onset times, Δt indicates difference in signal onset times (see Methods). **C.** Summary data for Δt measurements for methyl tiglate (MT) and M2MB. Bars indicate mean Δt; points are biological replicates; error bars show 95%CI. Δt = 302 ± 30 ms (mean ± SEM.) for MT (n = 5); 313 ± 5 ms (n =5) for M2MB. **D.** Schematic of model for simulating absorption and metabolism of inhaled odorant during respiration in the mouse olfactory epithelium. See Text for details. **E.** Dynamics of inhaled odorant (simulated methyl tiglate) concentration in the mucus compartment, with and without the presence of carboxylesterase enzyme, and acid metabolite product (tiglic acid; orange), following a single inhalation. Gray plot shows odorant concentration profile in the airway, used as input to the simulation. Metabolite concentration rises with a delay of ~ 100 ms and a slower decay. **F.** Simulated odorant and metabolite dynamics during repeated inhalations at 4 Hz, showing accumulation of metabolite in the mucus over the first several inhalations, with weak modulation by each successive inhalation. **G.** Inhalation-triggered average (ITA) of inhaled odorant and metabolite during successive inhalations, showing delayed and less-modulated dynamics of the metabolite. **H.** OSN response onsets (‘OSN-GCaMP8m’) to tiglic acid and its ester, aldehyde and alcohol derivatives, aligned to the first inhalation of each odorant. Responses to the derivatives are delayed relative to the acid, with tiglic alcohol showing the largest delay. Numbers indicate total odorant dilution. **I.** Summary data showing delays in onset latency for the ester, aldehyde and alcohol derivatives for the four identified glomeruli, relative to that of the acid measured in the same session. Data presented as mean ± 95% CI; points indicate biological replicates; n = 3 – 6 replicates per glomerulus. Δacid latency (emm ± SEM), esters: 98 ± 11 ms (t(18) = −8.7, *p* = 2E-7); aldehydes: 88 ± 11 ms (t(18) = −7.6, *p* = 1.5E-6); alcohols: 205 ± 38 ms (t(10) = −5.4, *p*=7E-4). Bonferroni-corrected pairwise comparisons between acids and their corresponding esters, aldehydes, and alcohols. ****p < 0.0001. **J.** Single-trial traces of iGluSnFR3 signals during presentation of tiglic acid and methyl tiglate in the awake mouse, taken from primary glomerulus. Top trace shows respiration signal; vertical lines indicate start of each inhalation during odorant presentation. Acid-evoked signals are strongly modulated by each inhalation, while ester signals are slower-onset and show weaker modulation. Full trial is shown in Fig. Extended Data Fig. S4C. **K.** Paired onset latencies in response to each primary acid odorant and its ester derivative, plotted separately for OSN-GCaMP8m and MT-iGluSnFR signals. Each paired point is a replicate, colors indicate identified glomerulus/primary odorant, as in (I). MT-iGluSnFR mean Δacid, 69 ± 9 ms, t(13) = −7.7; *p* = 3E-6). ****p < 0.0001. **L.** Inhalation-triggered average (ITA) traces of iGluSnFR3 signals during successive inhalations of tiglic acid and methyl tiglate, showing delayed inhalation-linked onset (defined by trough of ITA) and weaker modulation for the ester. From same session as in (J). **M.** Paired comparisons of ITA latency (left) and modulation index (right) measured from ITA traces for the acid and ester derivatives, plotted for each primary odorant, as in (K). Δacid latency, 128 ± 15 ms, t(13) = −8.4, *p* = 2E-6; Δacid MI, −0.36 ± 0.05, t(13) = 7.4, *p* = 6E-6; ****p < 0.0001. All statistical tests in the Figure use Linear mixed-effects model with pairwise comparisons of estimated marginal means (emm). See Methods.

**Figure 3.**
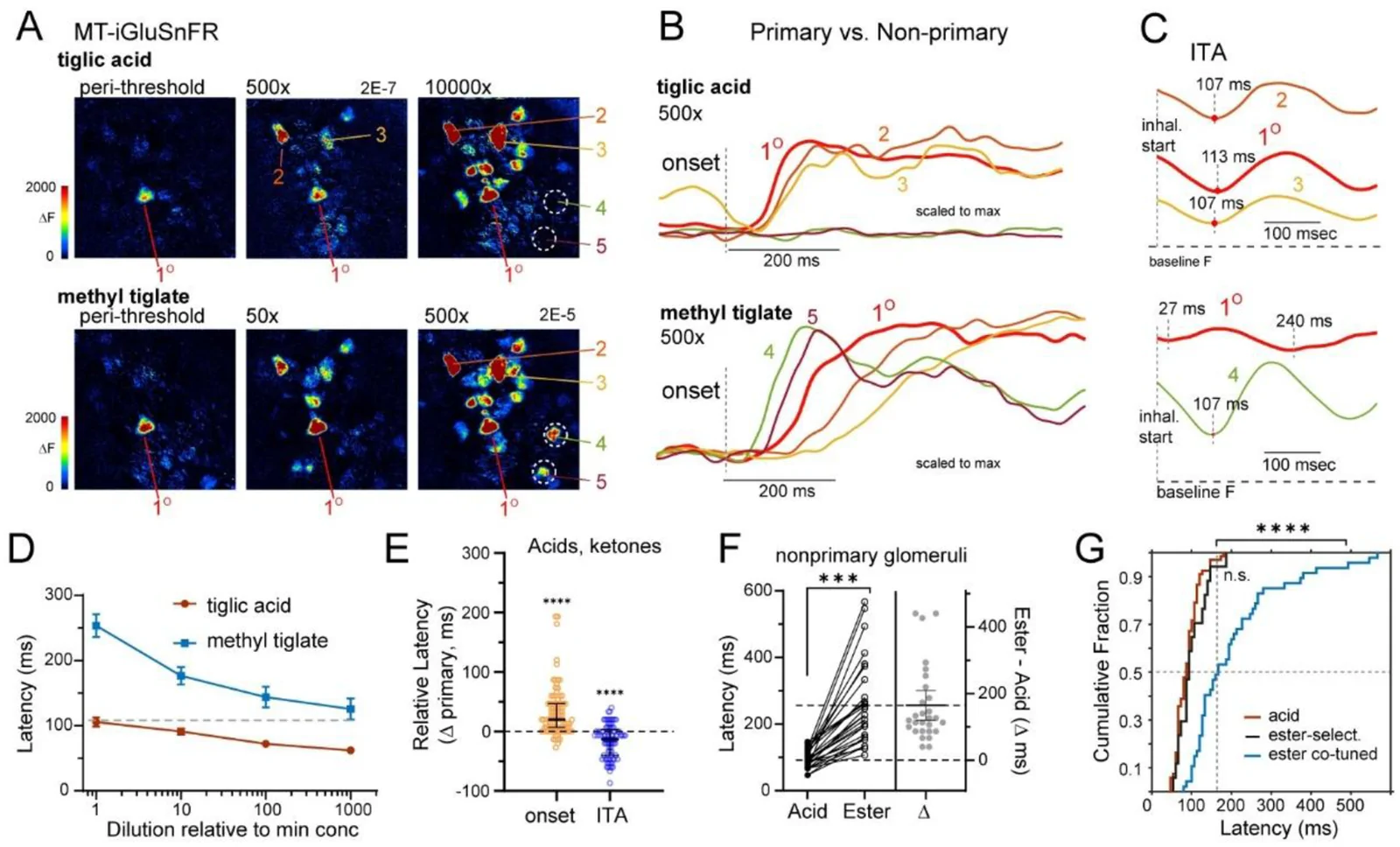
Odorant metabolism controls the timing of OSN inputs across odorant concentration and glomerular sensitivity. **A.** MT-iGLuSnFR3 response maps showing primary (i.e., most-sensitive) glomerulus for tiglic acid (TA, top row) and its ester derivative methyl tiglate (MT, bottom row) and recruitment of non-primary glomeruli at higher odorant concentrations. **B.** Overlay of inhalation-aligned response onsets in primary and non-primary (less-sensitive) glomeruli evoked by 500x threshold concentration of TA or MT. Top: Non-primary responses to TA are slightly delayed relative to the primary glomerulus. Bottom: Two non-primary glomeruli (ROIs 4 and 5) respond with shorter latency to the primary glomerulus, despite being ~500x less sensitive. **C.** ITA traces generated from the same trials as in (D). Top: Primary and non-primary glomeruli show near-identical latencies during successive inhalations of TA. Bottom: The shorter response latency in the non-primary ROI 4 persists during successive inhalations and is identical to that of TA-evoked ITA responses (compare to top traces). Note that MT ITA onset is delayed by greater than one respiratory cycle (dashed line at 240 ms), such that an early trough is apparent reflecting the response to the preceding inhalation (dashed line at 27 ms). **D.** MT-iGluSnFR onset latencies for TA and its ester derivative MT as a function of concentration, measured from replicates of the TA-sensitive identified glomerulus. Dashed line, mean latency for TA at near-threshold concentration, for reference. Points and error bars indicate mean ± SEM. **E.** Odorant onset (‘Onset’) and ITA latencies (‘ITA’) for all non-primary glomeruli, relative to the primary glomerulus measured in the same trials, recruited by suprathreshold concentrations of carboxylic acids or ketones. Onset latencies are slightly but significantly longer than their respective primary glomeruli (median Δprimary: 20 ms; interquartile range (IQR): 7 – 47 ms; V=3163, *p*= 1E-12; n = 96 glomeruli, 7 mice, 8 OBs, One-sample Wilcoxon Signed Ranks against zero). ITA latencies are slightly but significantly shorter (median Δprimary: −13 ms; IQR: −40 – 3 ms; V = 623, *p* = 8E-6, n = 85 glomeruli, 7 mice, 8 OBs). Test:. ****p < 1E-4. **F.** Paired comparison of onset latencies (MT-iGluSnFR) for each carboxylic acid and its ester derivative (3 acid-ester pairs), measured in ‘nonprimary’ glomeruli recruited at suprathreshold concentrations. Comparisons made at matched threshold-referenced dilutions (10x - 100x threshold). Right plot shows estimation plot of differences, error bars, 95%CI. Δacid = 197 ± 27 ms (mean ± SEM), t(27) = 7.6, *p* = 2E-4; Linear mixed-effects model followed by pairwise comparison between acids and esters using estimated marginal means; n = 28 glomeruli, 5 mice, 6 OBs. ***p < 1E-3. **G.** Cumulative distribution plots comparing onset latencies for acid-evoked responses in acid-sensitive glomeruli (‘acid’, n = 67), ester-evoked responses in the same glomeruli (‘ester co-tuned’, n = 47), and ester-evoked responses in ester-selective glomeruli (‘ester-select’, n = 17). There is no difference in latency distributions between ‘acid’ and ‘ester-select’ responses (D = 0.20, *P* = 0.62, Kolmogorov-Smirnov test), while ‘ester co-tuned’ latencies are significantly longer than ‘acid’ latencies (W=255, *P* = 3E-14, Mann-Whitney test). Data from 5 mice, 6 OBs. ****p < 1E-4.

**Figure 4.**
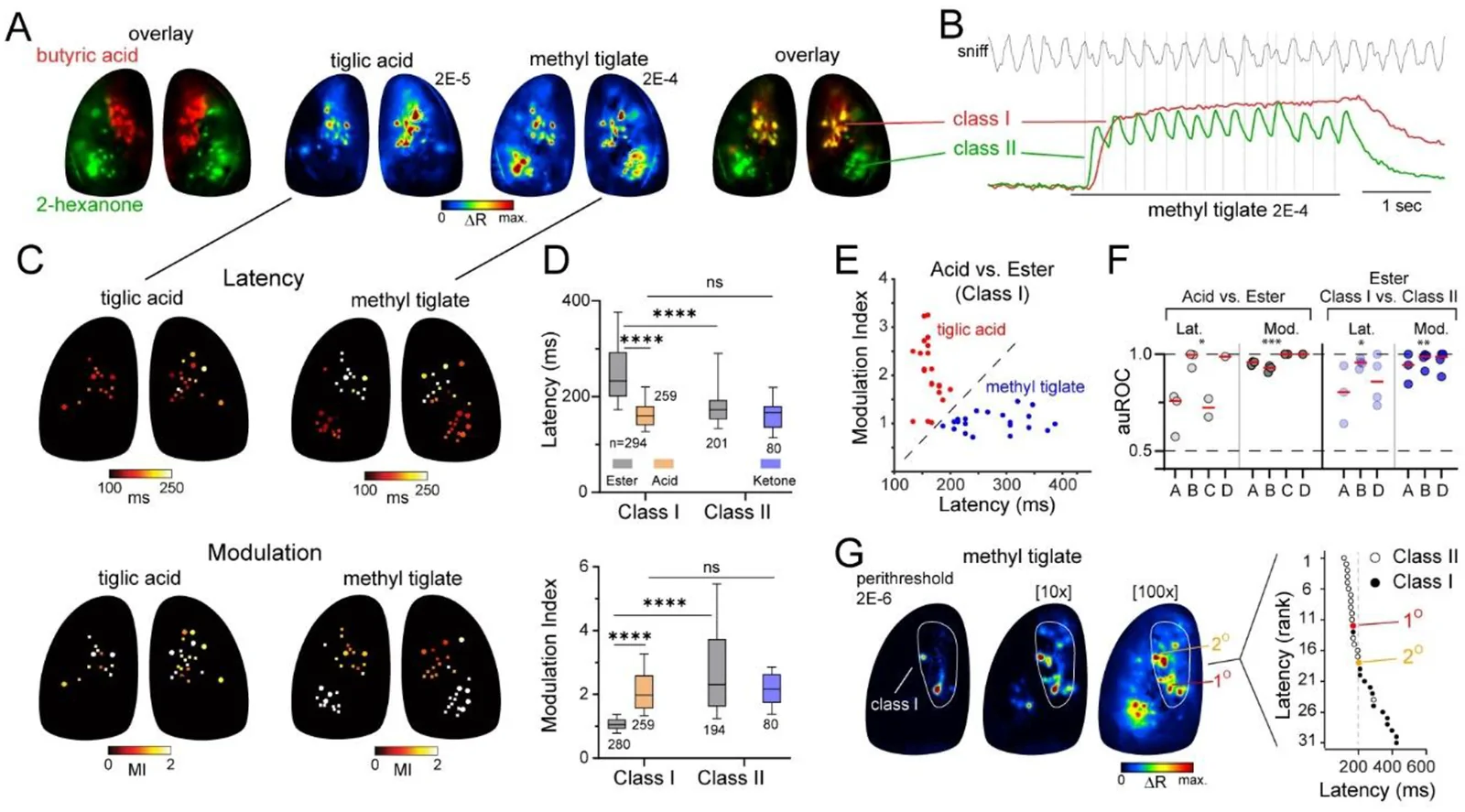
Inhalation-linked timing separates glomerular representations of inhaled versus metabolism-generated odorants. **A.** Widefield OSN-GCaMP8m response maps to suprathreshold concentrations of the acid TA and its ester derivative MT. Red-green overlay (right) shows near-complete overlap of TA and MT - responsive glomeruli in the anterior-medial OB and selective activation of caudal-lateral glomeruli by MT. Left-most map shows overlay (in the same mouse) of response to suprathreshold carboxylic acid (butyric acid) and ketone (2-hexanone), defining class I and class II-expressing OSN projection domains. **B.** GCaMP8m traces imaged from one glomerulus in the class I (red) and class II (green) domains, in response to MT in the same trial. Top trace shows mouse respiration, vertical lines indicate beginning of each inhalation. Note slower onset and lack of modulation in the class I - located glomerulus. **C.** Maps of glomeruli evoked by TA or MT (same data as in (A)), colored by onset latency (top) or MI (bottom), showing topography of each metric matching OR class-defined domains. **D.** Comparison of onset latency (top) or modulation index (MI, bottom) for ester or acid-evoked responses, grouped by glomerular location (class I domain or non-class I location). Response metrics for 2-hexanone (‘Ketone’), defining class II glomeruli, are included for reference. Box and whisker plots show median, interquartile and 10 – 90th percentile ranges. For ester-evoked responses, latencies are longer and MI is weaker for class I versus class II glomeruli (Latency: t(393) = 6.1, *p* = 3E-9; MI: t(198) = −13.3, *p* = 2E16). Among class I glomeruli, latencies are longer and MI is weaker for esters versus acids (Latency: t(528) = −11.3, *p* = 2E-16; MI: t(306) = 20.7, *p* = 2E-16). Latencies and MI are not different for responses to acids versus ketones (Latency: t(126.5) = −0.3, *p* = 0.76; MI: t(168) = 0.4, *p* = 0.7). Data from four acid-ester pairs, 7 OBs in 4 mice. Acids: tiglic acid, 2-methyl butyric acid, butyric acid, valeric acid; Esters: methyl tiglate, methyl-2-methyl butyrate, ethyl butyrate, methyl valerate. Unpaired t-tests, ****p < 1E-4. **E.** Modulation Index versus Latency plotted for glomeruli shown in (C). Points show responses of class I glomeruli to Acid (tiglic acid, red) or Ester (methyl tiglate, blue). Dashed line indicates plane separating each group, with perfect classification. See Fig. S7A for Class I vs. Class II classification. **F.** Classification performance scores (auROC) for specific acid-ester pairs (left) or specific esters (right). Points indicate auROC calculated from one OB. Red line, median. Asterisks indicate significant classification (auROC > 0.5) across all acid-ester replicates within a group. Paired acids vs. esters: Latency, t(2) = 4.4, *p* = 0.02; MI, t(2) = 25.1, *p* = 1.4E-3; Class I vs. Class II: Latency, t(1) = 8.3, *p* = 0.015; MI, t(1) = 27.5, *p* = 1.6E-3. Linear mixed-effects model with one-sided emms post hoc test (null = 0.5 auROC). Acid-ester pairs: A, butyric acid - ethyl butyrate; B, tiglic acid - methyl tiglate; C, valeric acid - methyl valerate; D, 2-methyl butyric acid - methyl 2-methyl butyrate. ***p < 1e-3, **p <.01, *p<0.05. **G.** Left: Widefield response maps to three concentrations of methyl tiglate (100-fold range) reveal widespread violations of sensitivity-timing relationships. The glomeruli most sensitive to MT (1°, 2°) are located within the class I domain. Right: Dot plot with ranked latencies of glomeruli activated by suprathreshold concentrations of MT, with 1° and 2° glomeruli highlighted. Filled or open dots indicate glomeruli in the class I or class II domains. Across 7 OBs in 4 mice, mean rank percentile of the primary glomerulus was 48 ± 4 %-ile (mean ± SD), raw rank: 12 ± 2 of 24 ± 5 ROIs).

**Figure 5.**
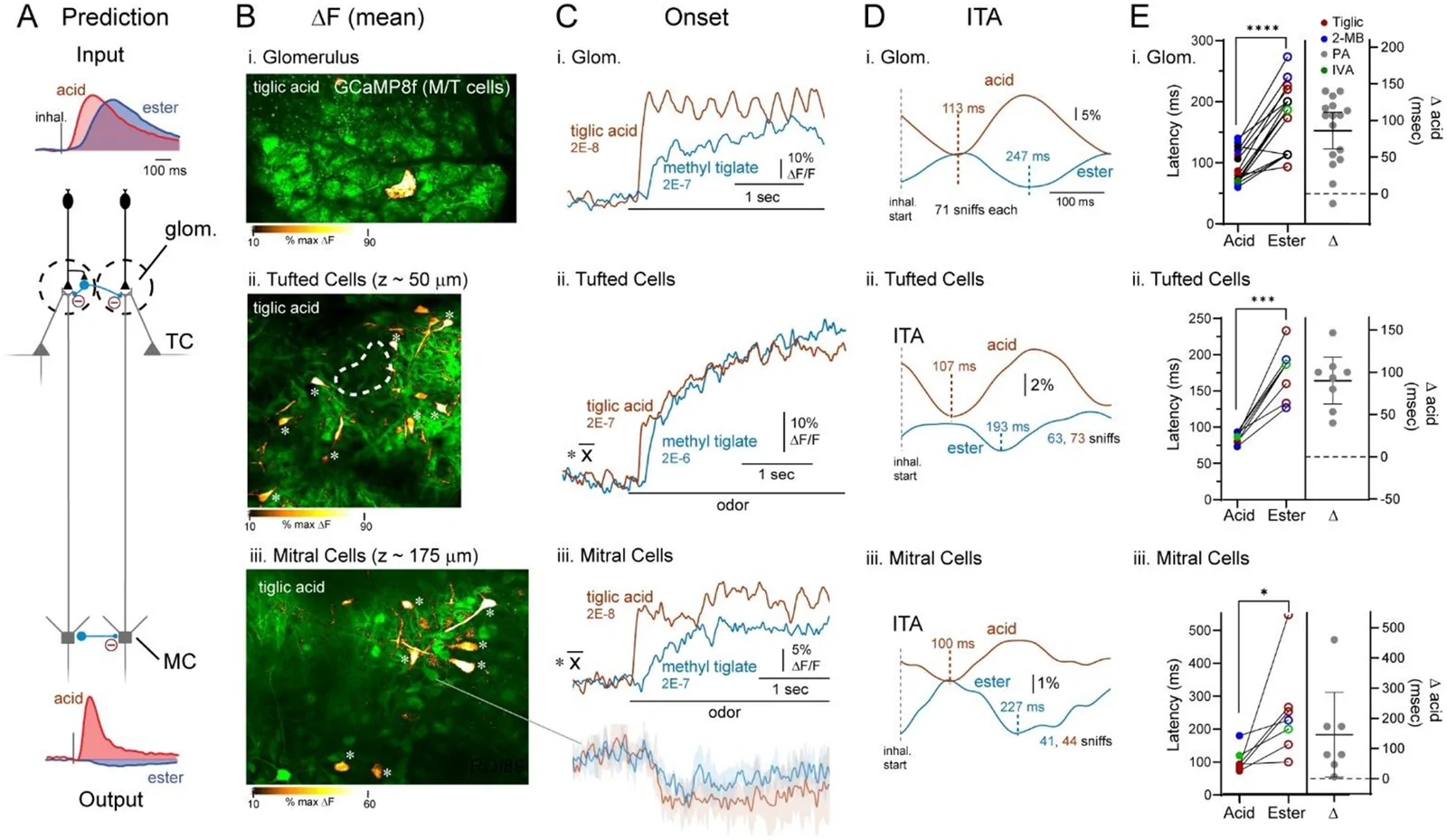
Delayed sensory inputs persist as delayed OB outputs among mitral and tufted cells. **A.** Schematic illustrating predicted suppression of delayed sensory inputs by inhibitory OB circuits and its appearance at different levels. **B.** MT-GCaMP8f signal overlays showing response (ΔF) to near-threshold tiglic acid (TA) imaged at the level of (i) M/T cell dendritic tufts; (ii) superficial TCs; and (iii) MCs. Signal overlays are scaled and clipped as indicated by the colorbar. All images are from the same OB. Asterisks indicate somata designated as daughter cells and averaged for subsequent analysis (see Text). **C.** Inhalation-aligned response onsets to TA and methyl tiglate (MT; 10x threshold concentration), showing delayed response to the ester for dendritic signals (i), TC somata (ii) and MC somata (iii). Bottom traces in (iii) show signal in one adjacent MC that is suppressed by both odorants. Shaded area, 95% CI across trials. **D.** Inhalation-triggered average (ITA) traces for successive inhalations of TA and MT, showing phase-delayed response waveforms in the ester-evoked response at all three levels. Same trials as (C). **E.** Paired latency measurements and difference estimation plots (mean ± 95%CI) for each primary acid and its ester derivative. Points of same color are biological replicates per glomerulus-primary odorant pair (indicated in legend). (i) Glomeruli, Δ acid (emm ± SEM) = 86 ± 12 ms, n = 16; t(15) = −7.4, *p* = 6E-6; (ii) TCs, Δ acid= 90 ± 12 ms, n = 8 parent glomeruli; t(7) = −7.7, *p* = 1E-4; (iii) MCs, Δ acid = 146 ± 57 ms, n = 7 parent glomeruli; t(6) = −2.5, *p* = 0.044. Linear mixed-effects model, post-hoc pairwise emms comparison. ****p < 1e-4, ***p < 0.001; *p < 0.05.

## Data Availability

Data used in all analyses will be made available by the authors on request. All custom analysis code is available via Github, at https://github.com/WachowiakLab.

## References

[1] Malnic B., Hirono J., Sato T. & Buck L. B. Combinatorial receptor codes for odors. Cell 96, 713–723 (1999). 10.1016/s0092-8674(00)80581-4 10089886

[2] Stanek J. J. & Morris J. B. The effect of inhibition of aldehyde dehydrogenase on nasal uptake of inspired acetaldehyde. Toxicol Sci 49, 225–231 (1999). 10.1093/toxsci/49.2.225 10416267

[3] Bogdanffy M. S. & Taylor M. L. Kinetics of nasal carboxylesterase-mediated metabolism of vinyl acetate. Drug Metab Dispos 21, 1107–1111 (1993). 7905391

[4] Takaoka N. Involvement of aldehyde oxidase in the metabolism of aromatic and aliphatic aldehyde-odorants in the mouse olfactory epithelium. Archives of Biochemistry and Biophysics 715, 109099 (2022). 10.1016/j.abb.2021.109099 34856193

[5] Spors H., Wachowiak M., Cohen L. B. & Friedrich R. W. Temporal dynamics and latency patterns of receptor neuron input to the olfactory bulb. J Neurosci 26, 1247–1259 (2006). 10.1523/JNEUROSCI.3100-05.2006 PMC6674558 16436612

[6] Fulton K. A., Zimmerman D., Samuel A., Vogt K. & Datta S. R. Common principles for odour coding across vertebrates and invertebrates. Nature Reviews Neuroscience 25, 453–472 (2024). 10.1038/s41583-024-00822-0 38806946

[7] Wilson C. D., Serrano G. O., Koulakov A. A. & Rinberg D. A primacy code for odor identity. Nat Commun 8, 1477 (2017). 10.1038/s41467-017-01432-4 PMC5684307 29133907

[8] Soelter J., Schumacher J., Spors H. & Schmuker M. Computational exploration of molecular receptive fields in the olfactory bulb reveals a glomerulus-centric chemical map. Scientific reports 10, 77 (2020). 10.1038/s41598-019-56863-4 PMC6952415 31919393

[9] Burton S. D. Mapping odorant sensitivities reveals a sparse but structured representation of olfactory chemical space by sensory input to the mouse olfactory bulb. eLife 11, e80470 (2022). 10.7554/eLife.80470 PMC9352350 35861321

[10] Ibarra-Soria X., Levitin M. O., Saraiva L. R. & Logan D. W. The Olfactory Transcriptomes of Mice. PLoS genetics 10, e1004593 (2014). 10.1371/journal.pgen.1004593 PMC4154679 25187969

[11] Olson M. J., Martin J. L., LaRosa A. C., Brady A. N. & Pohl L. R. Immunohistochemical localization of carboxylesterase in the nasal mucosa of rats. J Histochem Cytochem 41, 307–311 (1993). 10.1177/41.2.8419465 8419465

[12] Heydel J. M., Faure P. & Neiers F. Nasal odorant metabolism: enzymes, activity and function in olfaction. Drug Metab Rev 51, 224–245 (2019). 10.1080/03602532.2019.1632890 31203698

[13] Heydel J.-M. Odorant-Binding Proteins and Xenobiotic Metabolizing Enzymes: Implications in Olfactory Perireceptor Events. The Anatomical Record 296, 1333–1345 (2013). 10.1002/ar.22735 23907783

[14] Kornbausch N., Debong M. W., Buettner A., Heydel J.-M. & Loos H. M. Odorant Metabolism in Humans. Angewandte Chemie International Edition 61, e202202866 (2022). 10.1002/anie.202202866 PMC9541901 35522818

[15] Dahl A. R. & Hadley W. M. Nasal Cavity Enzymes Involved in Xenobiotic Metabolism: Effects on the Toxicity of Inhalants. Critical Reviews in Toxicology 21, 345–372 (1991). 10.3109/10408449109019571 1741949

[16] Thornton-Manning J. R. & Dahl A. R. Metabolic capacity of nasal tissue: interspecies comparisons of xenobiotic-metabolizing enzymes. Mutation Research - Fundamental and Molecular Mechanisms of Mutagenesis 380, 43–59 (1997). 10.1016/S0027-5107(97)00126-7 9385389

[17] Robert-Hazotte A. Nasal Odorant Competitive Metabolism Is Involved in the Human Olfactory Process. J Agric Food Chem 70, 8385–8394 (2022). 10.1021/acs.jafc.2c02720 35776896

[18] Shirai T. Functions of human olfactory mucus and age-dependent changes. Scientific reports 13, 971 (2023). 10.1038/s41598-023-27937-1 PMC9846672 36653421

[19] Bogdanffy M. S., Kee C. R., Hinchman C. A. & Trela B. A. Metabolism of dibasic esters by rat nasal mucosal carboxylesterase. Drug Metab Dispos 19, 124–129 (1991). 1673384

[20] Frederick C. B. Use of a hybrid computational fluid dynamics and physiologically based inhalation model for interspecies dosimetry comparisons of ester vapors. Toxicol Appl Pharmacol 183, 23–40 (2002). 10.1006/taap.2002.9451 12217639

[21] Niimura Y. & Nei M. Extensive Gains and Losses of Olfactory Receptor Genes in Mammalian Evolution. PLOS ONE 2, e708 (2007). 10.1371/journal.pone.0000708 PMC1933591 17684554

[22] Bozza T. Mapping of class I and class II odorant receptors to glomerular domains by two distinct types of olfactory sensory neurons in the mouse. Neuron 61, 220–233 (2009). 10.1016/j.neuron.2008.11.010 PMC3013286 19186165

[23] Billesbølle C. B. Structural basis of odorant recognition by a human odorant receptor. Nature 615, 742–749 (2023). 10.1038/s41586-023-05798-y PMC10580732 36922591

[24] Saito H., Chi Q., Zhuang H., Matsunami H. & Mainland J. D. Odor coding by a Mammalian receptor repertoire. Sci Signal 2, ra9 (2009). 10.1126/scisignal.2000016 PMC2774247 19261596

[25] Araneda R. C., Kini A. D. & Firestein S. The molecular receptive range of an odorant receptor. Nat Neurosci 3, 1248–1255 (2000). 10.1038/81774 11100145

[26] Nara K., Saraiva L. R., Ye X. & Buck L. B. A Large-Scale Analysis of Odor Coding in the Olfactory Epithelium. The Journal of Neuroscience 31, 9179 (2011). 10.1523/JNEUROSCI.1282-11.2011 PMC3758579 21697369

[27] Lee B. K. A principal odor map unifies diverse tasks in olfactory perception. Science 381, 999–1006 (2023). 10.1126/science.ade4401 PMC11898014 37651511

[28] Wesson D. W., Carey R. M., Verhagen J. V. & Wachowiak M. Rapid Encoding and Perception of Novel Odors in the Rat. PLoS Biology 6, e82 (2008). 10.1371/journal.pbio.0060082 PMC2288628 18399719

[29] Rinberg D., Koulakov A. & Gelperin A. Speed-accuracy tradeoff in olfaction. Neuron 51, 351–358 (2006). 10.1016/j.neuron.2006.07.013 16880129

[30] Abraham N. M. Maintaining accuracy at the expense of speed: stimulus similarity defines odor discrimination time in mice. Neuron 44, 865–876 (2004). 10.1016/j.neuron.2004.11.017 15572116

[31] Uchida N. & Mainen Z. F. Speed and accuracy of olfactory discrimination in the rat. Nat Neurosci 6, 1224–1229 (2003). 10.1038/nn1142 14566341

[32] Jiang J. & Zhao K. Airflow and nanoparticle deposition in rat nose under various breathing and sniffing conditions: a computational evaluation of the unsteady effect. J Aerosol Sci 41, 1030–1043 (2010). 10.1016/j.jaerosci.2010.06.005 PMC2976565 21076632

[33] Marvin J. S. Stability, affinity, and chromatic variants of the glutamate sensor iGluSnFR. Nat Methods 15, 936–939 (2018). 10.1038/s41592-018-0171-3 PMC6394230 30377363

[34] Aggarwal A. Glutamate indicators with improved activation kinetics and localization for imaging synaptic transmission. Nature Methods 20, 925–934 (2023). 10.1038/s41592-023-01863-6 PMC10250197 37142767

[35] Moran A. K., Eiting T. P. & Wachowiak M. Dynamics of Glutamatergic Drive Underlie Diverse Responses of Olfactory Bulb Outputs In Vivo. eNeuro 8, ENEURO.0110–0121.2021 (2021). 10.1523/eneuro.0110-21.2021 PMC8059884 33795414

[36] Moran A. K., Eiting T. P. & Wachowiak M. Circuit Contributions to Sensory-Driven Glutamatergic Drive of Olfactory Bulb Mitral and Tufted Cells During Odorant Inhalation. Front Neural Circuits 15, 779056 (2021). 10.3389/fncir.2021.779056 PMC8578712 34776878

[37] von der Weid B. Large-scale transcriptional profiling of chemosensory neurons identifies receptor-ligand pairs in vivo. Nature Neuroscience 18, 1455–1463 (2015). 10.1038/nn.4100 26322926

[38] Karadas M., Gill J. V., Ceballo S., Shoham S. & Rinberg D. Rapid temporal processing in the olfactory bulb underlies concentration-invariant odor identification and signal decorrelation. Nat Neurosci (2026). 10.1038/s41593-026-02250-y PMC13156029 41981338

[39] Cichy A., Shah A., Dewan A., Kaye S. & Bozza T. Genetic Depletion of Class I Odorant Receptors Impacts Perception of Carboxylic Acids. Curr Biol 29, 2687–2697 e2684 (2019). 10.1016/j.cub.2019.06.085 PMC8049193 31378611

[40] St. Angelo A. J., Vercellotti J., Jacks T. & Legendre M. Lipid oxidation in foods. Critical Reviews in Food Science and Nutrition 36, 175–224 (1996). 10.1080/10408399609527723 8744604

[41] Ng K.-S., Bambace M. F. & Schwab C. Microbially produced short-chain carboxylic acids are ancient food biopreservatives with complex mode of action. Current Opinion in Food Science 52, 101066 (2023). 10.1016/j.cofs.2023.101066

[42] Kobayakawa K. Innate versus learned odour processing in the mouse olfactory bulb. Nature 450, 503–508 (2007). 10.1038/nature06281 17989651

[43] Beekwilder J. Functional characterization of enzymes forming volatile esters from strawberry and banana. Plant Physiol 135, 1865–1878 (2004). 10.1104/pp.104.042580 PMC520758 15326278

[44] Ceccoli R. D., Bianchi D. A., Zocchi S. B. & Rial D. V. Mapping the field of aroma ester biosynthesis: A review and bibliometric analysis. Process Biochemistry 146, 587–600 (2024). 10.1016/j.procbio.2024.10.002

[45] Chong E. Manipulating synthetic optogenetic odors reveals the coding logic of olfactory perception. Science 368 (2020). 10.1126/science.aba2357 PMC8237706 32554567

[46] Giaffar H., Shuvaev S., Rinberg D. & Koulakov A. A. The primacy model and the structure of olfactory space. PLoS Comput Biol 20, e1012379 (2024). 10.1371/journal.pcbi.1012379 PMC11423968 39255274

[47] Hopfield J. J. Pattern recognition computation using action potential timing for stimulus representation. Nature 376, 33–36 (1995). 10.1038/376033a0 7596429

[48] Gill J. V., Karadas M., Shoham S. & Rinberg D. Temporal sequence geometry enables odor recognition and generalization. bioRxiv (2026). 10.64898/2026.01.20.700611

[49] Bolding K. A. & Franks K. M. Recurrent cortical circuits implement concentration-invariant odor coding. Science 361 (2018). 10.1126/science.aat6904 PMC6492549 30213885

[50] Stokes C. C. A. & Isaacson J. S. From Dendrite to Soma: Dynamic Routing of Inhibition by Complementary Interneuron Microcircuits in Olfactory Cortex. Neuron 67, 452–465 (2010). 10.1016/j.neuron.2010.06.029 PMC2922014 20696382

[51] Blankenship M. L., Grigorova M., Katz D. B. & Maier J. X. Retronasal Odor Perception Requires Taste Cortex, but Orthonasal Does Not. Curr Biol 29, 62–69 e63 (2019). 10.1016/j.cub.2018.11.011 PMC6604050 30581018

[52] Mori K. & Sakano H. Neural Circuitry for Stress Information of Environmental and Internal Odor Worlds. Frontiers in Behavioral Neuroscience Volume 16 - 2022 (2022). 10.3389/fnbeh.2022.943647 PMC9245520 35783233

[53] Ye Y. Decomposition of an odorant in olfactory perception and neural representation. Nature Human Behaviour 8, 1150–1162 (2024). 10.1038/s41562-024-01849-0 38499771

[54] Chee-Ruiter C. W. J. The Biological Sense of Smell: Olfactory Search Behavior and a Metabolic View for Olfactory Perception PhD thesis, California Institute of Technology, (2000).

[55] Qian W. W. Metabolic activity organizes olfactory representations. eLife 12, e82502 (2023). 10.7554/eLife.82502 PMC10154027 37129358

[56] Yu C. R. Spontaneous Neural Activity Is Required for the Establishment and Maintenance of the Olfactory Sensory Map. Neuron 42, 553–566 (2004). 10.1016/s0896-6273(04)00224-7 15157418

[57] Wekselblatt J. B., Flister E. D., Piscopo D. M. & Niell C. M. Large-scale imaging of cortical dynamics during sensory perception and behavior. Journal of Neurophysiology 115, 2852–2866 (2016). 10.1152/jn.01056.2015 PMC4922607 26912600

[58] Short S. M. & Wachowiak M. Temporal Dynamics of Inhalation-Linked Activity across Defined Subpopulations of Mouse Olfactory Bulb Neurons Imaged In Vivo. eNeuro 6, ENEURO.0189–0119.2019 (2019). 10.1523/ENEURO.0189-19.2019 PMC6597857 31209151

[59] Nakayama H., Gerkin R. C. & Rinberg D. A behavioral paradigm for measuring perceptual distances in mice. Cell Rep Methods 2, 100233 (2022). 10.1016/j.crmeth.2022.100233 PMC9243525 35784646

[60] Burton S. D., Wipfel M., Guo M., Eiting T. P. & Wachowiak M. A Novel Olfactometer for Efficient and Flexible Odorant Delivery. Chem Senses 44, 173–188 (2019). 10.1093/chemse/bjz005 PMC6410398 30657873

[61] Cometto-Muñiz J. E., Cain W. S. & Abraham M. H. Quantification of Chemical Vapors in Chemosensory Research. Chemical Senses 28, 467–477 (2003). 10.1093/chemse/28.6.467 12907584

[62] Jennings L., Williams E., Caton S., Avlas M. & Dewan A. Estimating the relationship between liquid- and vapor-phase odorant concentrations using a photoionization detector (PID)-based approach. Chemical Senses 48, bjac038 (2023). 10.1093/chemse/bjac038 PMC10208748 36571813

[63] Mainland J. D. The missense of smell: functional variability in the human odorant receptor repertoire. Nature Neuroscience 17, 114–120 (2014). 10.1038/nn.3598 PMC3990440 24316890

[64] Zhang Y., Pan Y., Matsunami H. & Zhuang H. Live-cell Measurement of Odorant Receptor Activation Using a Real-time cAMP Assay. J Vis Exp (2017). 10.3791/55831 PMC5752358 28994818

[65] Ikegami K. Structural instability and divergence from conserved residues underlie intracellular retention of mammalian odorant receptors. Proc Natl Acad Sci U S A 117, 2957–2967 (2020). 10.1073/pnas.1915520117 PMC7022149 31974307

[66] Durant J. L., Leland B. A., Henry D. R. & Nourse J. G. Reoptimization of MDL Keys for Use in Drug Discovery. Journal of Chemical Information and Computer Sciences 42, 1273–1280 (2002). 10.1021/ci010132r 12444722

[67] Boichot V. Characterization of human oxidoreductases involved in aldehyde odorant metabolism. Scientific reports 13, 4876 (2023). 10.1038/s41598-023-31769-4 PMC10039900 36966166

[68] Wachowiak M., Dewan A., Bozza T., O’Connell T. F. & Hong E. J. Recalibrating Olfactory Neuroscience to the Range of Naturally Occurring Odor Concentrations. The Journal of Neuroscience 45, e1872242024 (2025). 10.1523/jneurosci.1872-24.2024 PMC11884396 40044450

[69] Hagberg A., Swart P. J. & Schult D. A. (Los Alamos National Laboratory (LANL), United States, 2008).

[70] Robert-Hazotte A. Ex vivo real-time monitoring of volatile metabolites resulting from nasal odorant metabolism. Scientific reports 9, 2492 (2019). 10.1038/s41598-019-39404-x PMC6385289 30792537

[71] Kay S. M. Funamentals of Statistical Signal Processing, Volume 2: Detection Theory. Vol. 2 (Prentice Hall, 1998).

[72] Scott J. W., Sherrill L., Jiang J. & Zhao K. Tuning to Odor Solubility and Sorption Pattern in Olfactory Epithelial Responses. The Journal of Neuroscience 34, 2025 (2014). 10.1523/JNEUROSCI.3736-13.2014 PMC3913860 24501345

[73] Kim K. & Zhao K. A Nasal Aerodynamics Perspective of Retronasal Olfaction: Rodents vs. Humans. Chemosens Percept 15, 124–134 (2022). 10.1007/s12078-022-09300-2 PMC10168102 37192933

[74] Wu Z., Jiang J., Lischka F. W. & Zhao K. Is the mouse nose a miniature version of a rat nose? A computational comparative study. Comput Methods Programs Biomed 254, 108282 (2024). 10.1016/j.cmpb.2024.108282 PMC12125470 38878359

[75] Kurtz D. B., Zhao K., Hornung D. E. & Scherer P. Experimental and numerical determination of odorant solubility in nasal and olfactory mucosa. Chem Senses 29, 763–773 (2004). 10.1093/chemse/bjh079 15574812

[76] Wilke C. R. & Chang P. Correlation of diffusion coefficients in dilute solutions. AIChE Journal 1, 264–270 (1955). 10.1002/aic.690010222

[77] Corley R. A. Comparative Risks of Aldehyde Constituents in Cigarette Smoke Using Transient Computational Fluid Dynamics/Physiologically Based Pharmacokinetic Models of the Rat and Human Respiratory Tracts. Toxicological Sciences 146, 65–88 (2015). 10.1093/toxsci/kfv071 PMC4476461 25858911

[78] Pachitariu M. Suite2p: beyond 10,000 neurons with standard two-photon microscopy. bioRxiv, 061507 (2017). 10.1101/061507

[79] Bates D., Mächler M., Bolker B. & Walker S. Fitting linear mixed-effects models using lme4. Journal of Statistical Software 67, 1–48 (2015). 10.18637/jss.v067.i01

