# Supplementary material for: Rapid nasal metabolism organizes the neural representation of odors
Source: Res Sq. 2026 Sep 4:rs.3.rs-10695342. Preprint. [Version 1] doi: 10.21203/rs.3.rs-10695342/v1 (PMC13684750; doi:10.21203/rs.3.rs-10695342/v1)
Supplement: Supplementary file 1 [file NIHPPRS10695342V1-supplement-1.pdf]

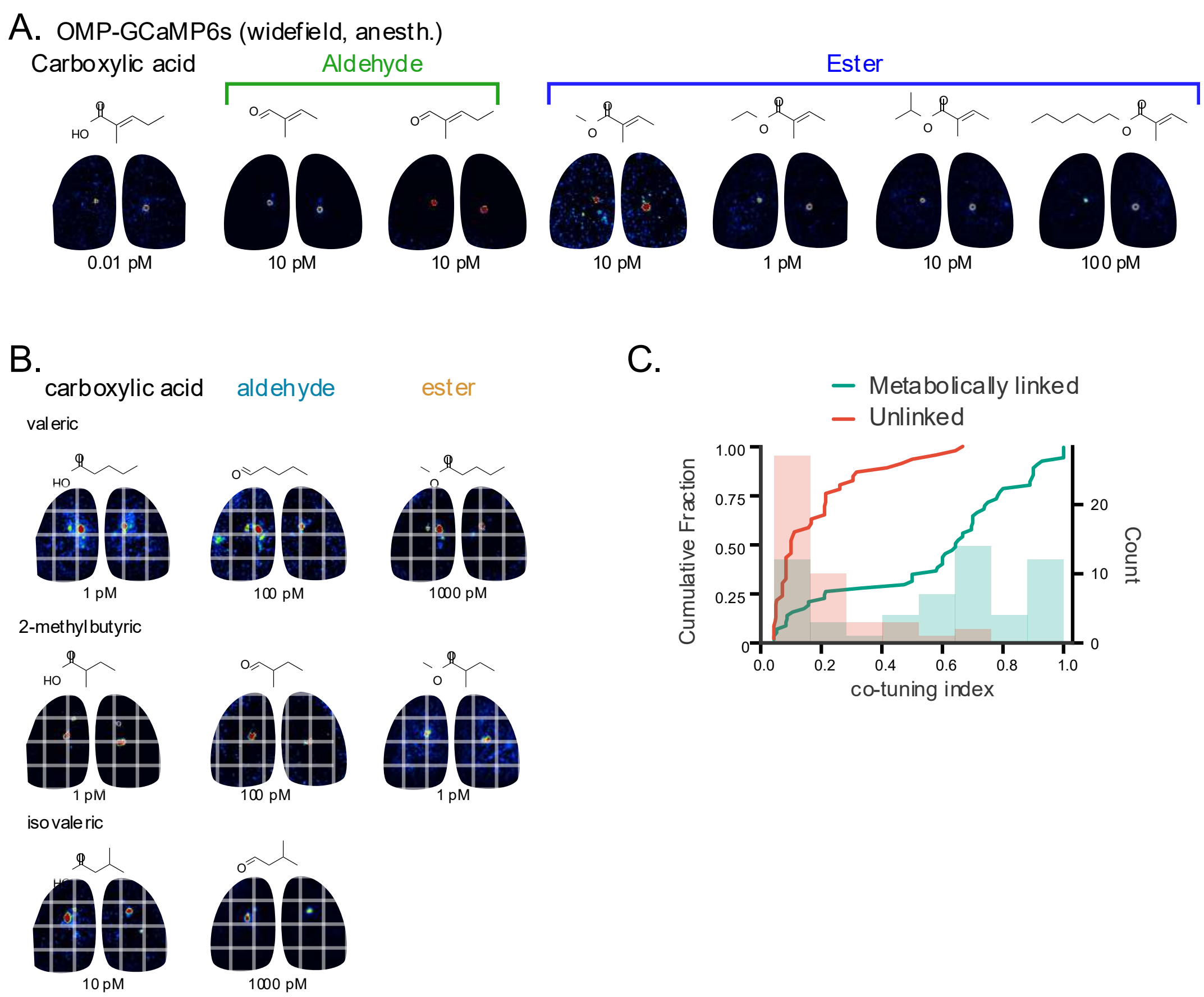

Extended Data Figure 1 (related to Figure 1). Sensitive co-tuning of glomeruli among carboxylic acids and their ester and aldehyde derivatives linked by nasal metabolism.

A. OMP-GCaMP6s odorant response maps showing potent and selective activation of the same glomerulus with primary sensitivity to 2-methyl-2-pentenoic acid and an array of aldehyde and ester derivatives. Data from [9].

B. Epifluorescence response maps of OSN inputs (OMP-IRES-tTA x tetO-GCaMP6s) showing near-identical selective activation of dorsal glomeruli to three additional carboxylic acids and their ester and aldehyde derivatives, at estimated delivered concentrations shown. Data from [9]. Compare to Fig. 1C.

C. Distribution of co-tuning metrics for the odorants shown in Fig. 1D, plotted separately for odorant pairs linked by CES or AOX/ALDH metabolism (green, 'Linked') or not (red, 'Unlinked'). Non co-tuned odorant pairs are omitted.

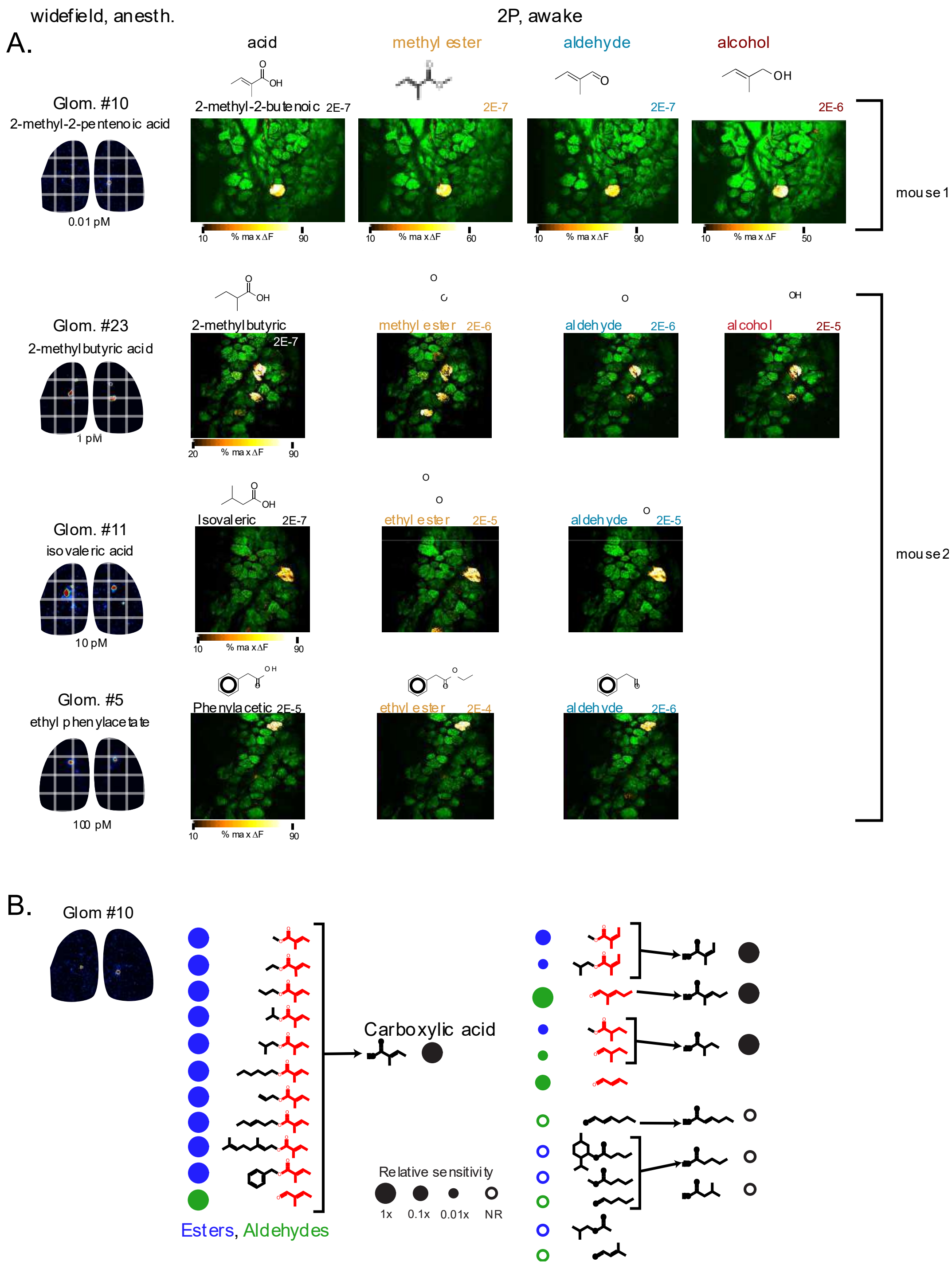

Extended Data Figure 2 (related to Figure 1). Sensitive co-tuning to carboxylic acids and derivatives for functionally-identified glomeruli imaged in awake mice.

A. Two-photon response maps of OSN inputs (OMP-Cre x TIGRE2-GCaMP8m) to each of four functionally-identified glomeruli, numbered and using identification criteria from [9], evoked by the

primary acid and its ester, aldehyde and (for A and B) alcohol derivatives. Reference widefield image from [9] showing singular activation is included for each (left). Numbers next to odorant name indicate total odorant dilution (liquid dilution x vapor-phase dilution; see Methods). Maps show ΔF overlaid on mean fluorescence, clipped and scaled as indicated.

B. Relative sensitivities of the functionally-identified glomerulus (glom. #10) to 30 odorants, shown with odorant structures and metabolic relationships, including 21 effective odorants plotted in Fig. 1F. Widefield image (from (A)) is shown for reference. Sensitivity is expressed relative to the estimated reference concentration for its primary odorant, tiglic acid, of 0.1 nM (see Methods). Each odorant was screened using a serial 10-fold concentration series beginning at this estimated concentration; circles indicate lowest relative concentration eliciting a response. Open circles indicate no response at 100x reference concentration (10 nM). Colors indicate odorant class and substrate for CEs or AOX/ALDH, respectively; brackets group odorants generating the same carboxylic acid metabolite. Data compiled from 6 mice (12 OBs): OMP-IRES-Cre x TIGRE2-GCaMP8m (n = 4);OMP-IRES-tTA x tetO-GCaMP6s (n = 2).

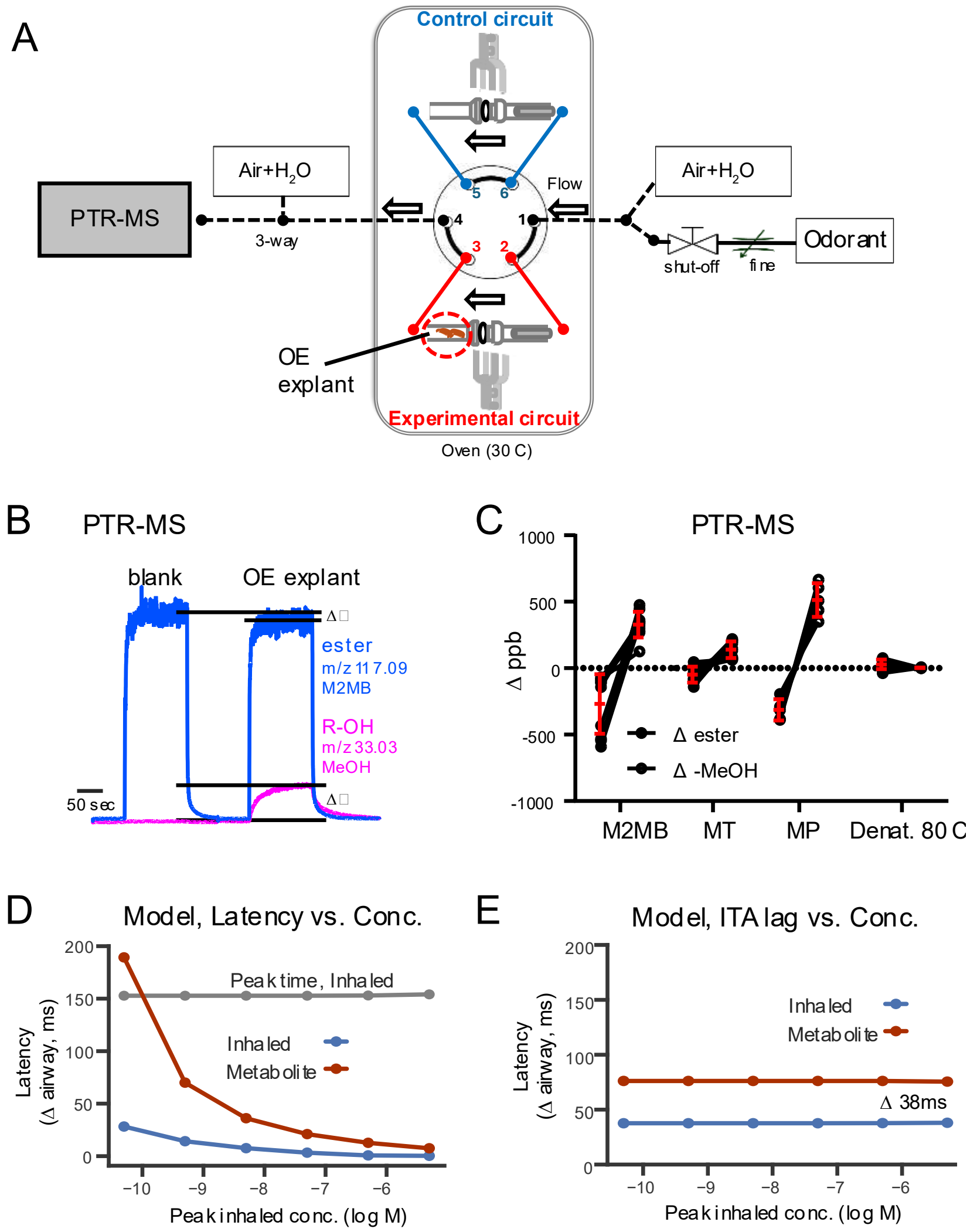

Extended Data Figure 3 (related to Figure 2). Additional details of ex vivo PTR-MS measurements and epithelial PBPK metabolism model predictions.

A. Detailed schematic of setup for time-resolved measurement of ester metabolism by olfactory mucosa using PTR-MS. Air flow is from right to left and follows either a control circuit (blue) or an experimental circuit (red) containing the mucosa explant before travelling to the PTR-ToF-MS detector.

B. PTR-MS signal for the ester methyl-2-methyl butyrate (M2MB) and its methanol hydrolysis product, measured with and without olfactory mucosa present. In the presence of tissue, the methanol signal

(pink) rapidly rises above blank values and eventually plateaus, while the M2MB signal is reduced relative to the no-tissue condition, consistent with ester metabolism.

C. Summary of reductions in ester signal amplitude and concomitant increases in methanol (MeOH) signal amplitude for olfactory mucosa flow, relative to control flow circuit, for different methyl esters tested. M2MB, methyl2-methyl butyrate; MT, methyl tiglate; MP, methyl propionate; Denat., Explant denatured by heat treatment to 80 C (see Methods).

D. Model-predicted onset latency for parent odorant and metabolite product as a function of peak inhaled concentration. Latency was defined as the time from inhaled odorant onset to a mucus concentration level of 1E-10 M. Gray trace shows time of peak mucus concentration for the inhaled odorant. Blue plot, latency for inhaled odorant (simulated methyl tiglate); Orange, latency for acid metabolite.

E. Model-predicted onset latency for parent odorant and metabolite product during successive inhalations, defined as trough of inhalation-triggered average (ITA) trace, as a function of peak inhaled concentration. In contrast to initial onset latencies, latencies for successive inhalations remain constant across concentration, with a Δt of 38 ms between parent and product.

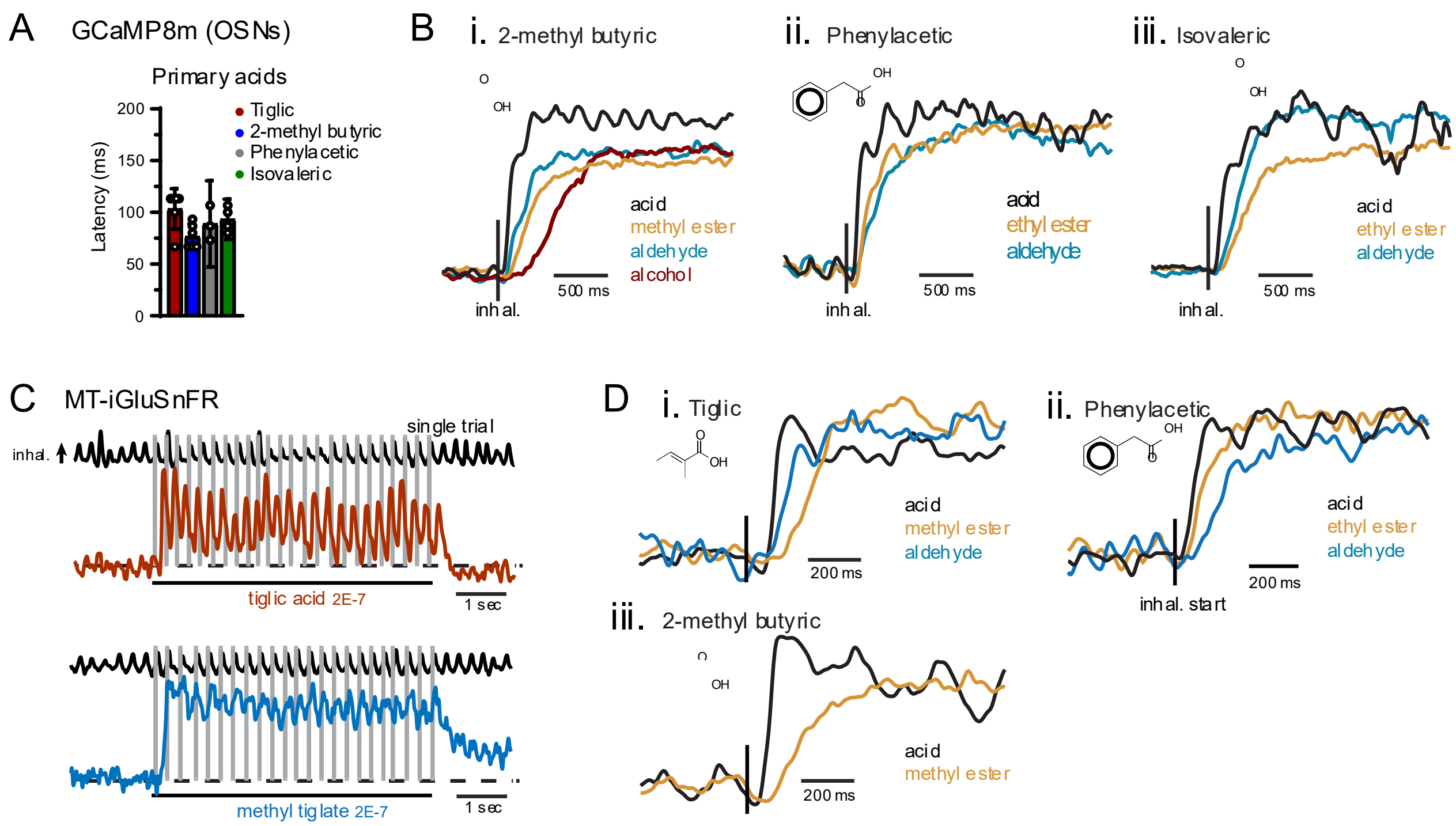

Extended Data Figure 4, related to Figure 2. Onset latency differences are consistent across different functionally-identified glomeruli and activity reporters.

A. Summary data showing similar onset latencies in each of the four functionally-identified glomeruli to their respective primary carboxylic acid odorant, measured with OSN-GCaMP8m signals. Bars and error bars indicate mean ±95%CI. Mean latencies (n replicates) for tiglic, 2-methyl butyric, phenylacetic and isovaleric: 103 (6), 77 (6), 89 (3) and 93 (4) ms, respectively.

B. Traces showing OSN response onsets (OSN-GCaMP8m) to the primary acid and its ester, aldehyde and alcohol derivatives aligned to the first inhalation of each odorant and averaged across 3 - 6 trials for glomeruli identified by their primary sensitivity to 2-methyl butyric acid (i), phenylacetic acid (ii) and isovaleric acid (iii). Traces taken from same trials and dilutions as for ΔF maps in Fig. S2A.

C. Full single-trial trace of iGluSnFR3 signals during presentation of tiglic acid and methyl tiglate in the awake mouse; same trial as in Fig. 2J. Top trace, respiration signal; vertical lines indicate start of each inhalation during odorant presentation.

D. Inhalation-aligned average traces of MT-iGluSnFR response onsets for tiglic acid (i), phenylacetic acid (ii) and 2-methyl butyric acid (iii) and their ester and aldehyde derivatives, taken from their respective primary glomeruli as in (B).

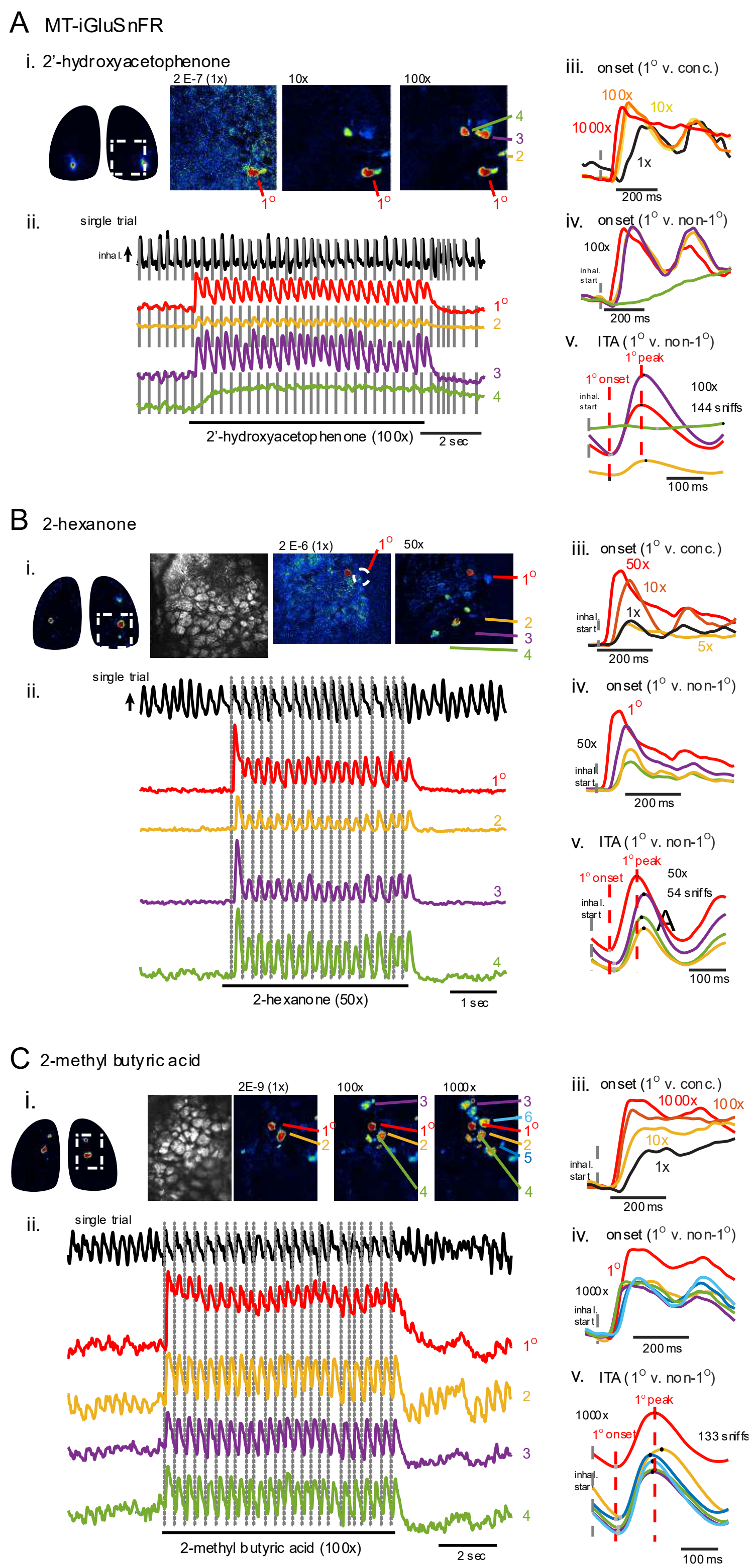

Extended Data Figure 5 (related to Fig. 3). Concentration- and sensitivity-timing relationships for additional functionally-identified glomeruli.

A-C. Response maps and inhalation-linked timing for MT-iGluSnFR3 signals in primary (i.e., most-sensitive) and recruited (non-primary) glomeruli for 2-hexanone (A), 2'-OH acetophenone (B) and 2-methyl butyric acid (C), plotted as shown in Fig. 3,A-C. (i) Widefield OMP-GCaMP6s response map showing singular activation, taken from Burton et al. [9]. (ii) Single trial of odorant presentation with traces from primary and select non-primary glomeruli, with respiration trace (top) and inhalation times (dashed line). (iii) Inhalation-aligned response onset for primary glomerulus evoked by increasing odorant concentrations, referenced to near-threshold concentration (1x). (iv). Inhalation-aligned response onsets in primary and non-primary glomeruli evoked by suprathreshold odorant concentration. (v). Inhalation-triggered (ITA) traces generated from the same glomeruli and suprathreshold trials as in (iv).

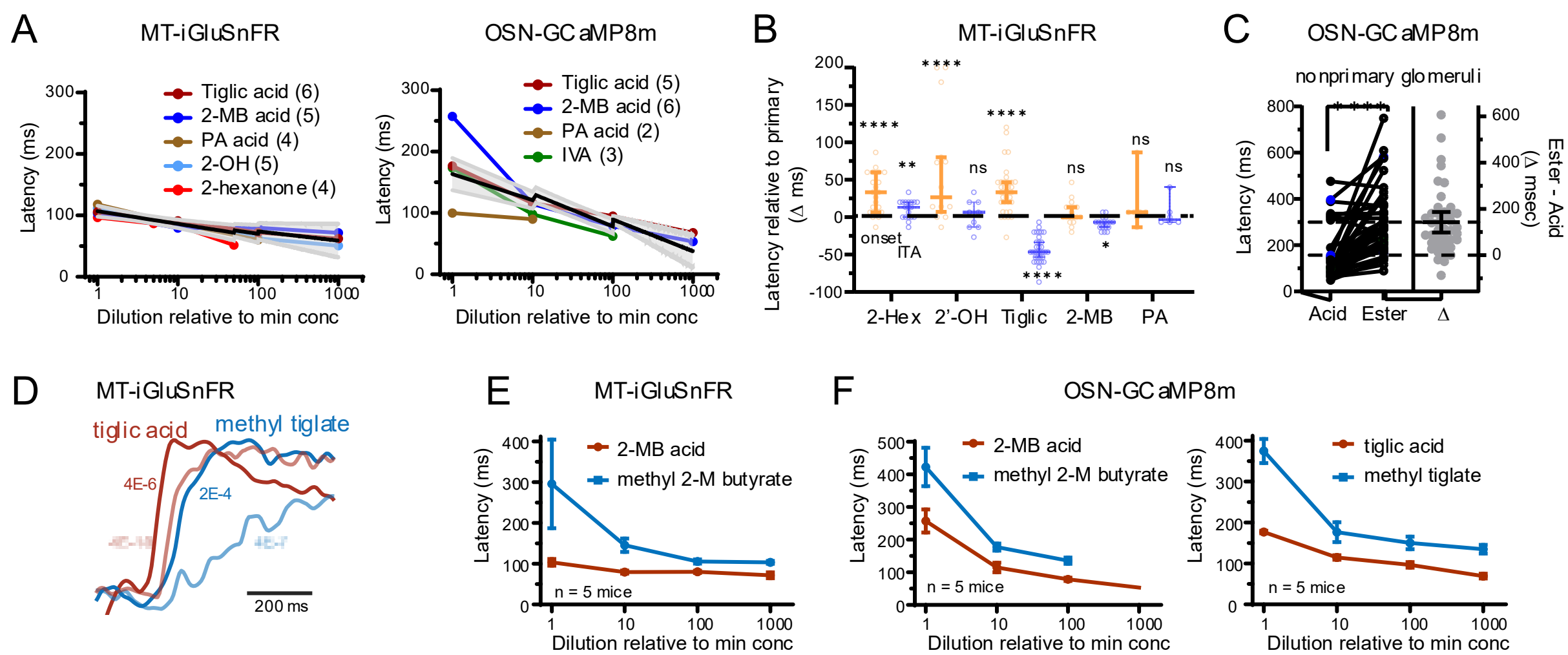

Extended Data Figure 6 (related to Fig. 3). Additional impacts of odorant concentration and glomerular sensitivity on inhalation-linked response latencies.

A. Onset latency versus concentration measured with MT-iGLuSnFR (left) or OMP-GCaMP8m (right) for the primary acid or ketone of identified glomeruli. Concentration values are referenced to the near-threshold dilution for each odorant. Colored plots show mean latencies across biological replicates (N shown in Legend, inset) for each glomerulus/primary odorant (plot legend indicates n for each). Solid black plot shows fit to the mean plots; shaded area indicates 95% confidence interval of fit. For MT-iGLuSnFR, fitted slope values (Δms per ten-fold Δconc) are: Tiglic acid: 15; 2-MB acid: 10; PA acid: 30; 2'-OH acetophenone (2-OH): 21; 2-hexanone: 28. For OMP-GCaMP8m, slope values are: Tiglic acid: 24; 2-MB acid: 27; Isovaleric acid (IVA): 36. PA acid was only tested at two concentrations.

B. Odorant onset and ITA latencies for non-primary glomeruli recruited by suprathreshold concentrations of carboxylic acids or ketones, relative to the primary glomerulus, grouped by odorant. Same data as in Fig. 3E. Data show variability in the magnitude of sensitivity-timing relationships for different odorants. One-way ANOVA, onset latency: $p < 2E-16$, $F(4,80) = 47.7$; ITA latency: $p = 0.002$; $F(4,91) = 4.5$. Asterisks indicate statistical comparison to zero (same latency as primary glom.). ****p <

1e-4, ***p < .001, **p < .01, *p<.05, Emmeans post-hoc test, Tukey adjustment for multiple comparisons.

C. Paired comparison of OMP-GCaMP8m onset latencies for each carboxylic acid and its ester derivative, measured in 'nonprimary' glomeruli, as in Fig. 3G. Right, estimation plot of differences, error bars, 95%CI. Δacid = 143 ± 22 ms, (mean ± SEM, n = 42 glomeruli), t(41) = -6.5, *p*=8E-8; Linear mixed-effects model followed by pairwise comparison of estimated marginal means; ****p < 1E-4.

D. Example traces showing inhalation-aligned responses to tiglic acid (TA, red) and methyl tiglate (MT, blue) presented at near-threshold and suprathreshold (1000 - 10000x) concentrations. Note that MT responses are slower than TA responses at all concentrations.

E. MT-iGluSnFR onset latencies for 2-methyl butyric acid and its ester derivative (methyl-2-methyl butyrate) as a function of concentration. Points indicate mean, error bars are SEM.

F. OMP-GCaMP8m onset latencies for 2-methyl butyric (left) and tiglic acid (right) and their respective ester derivatives, plotted as in (D), showing consistently longer latencies for the ester.

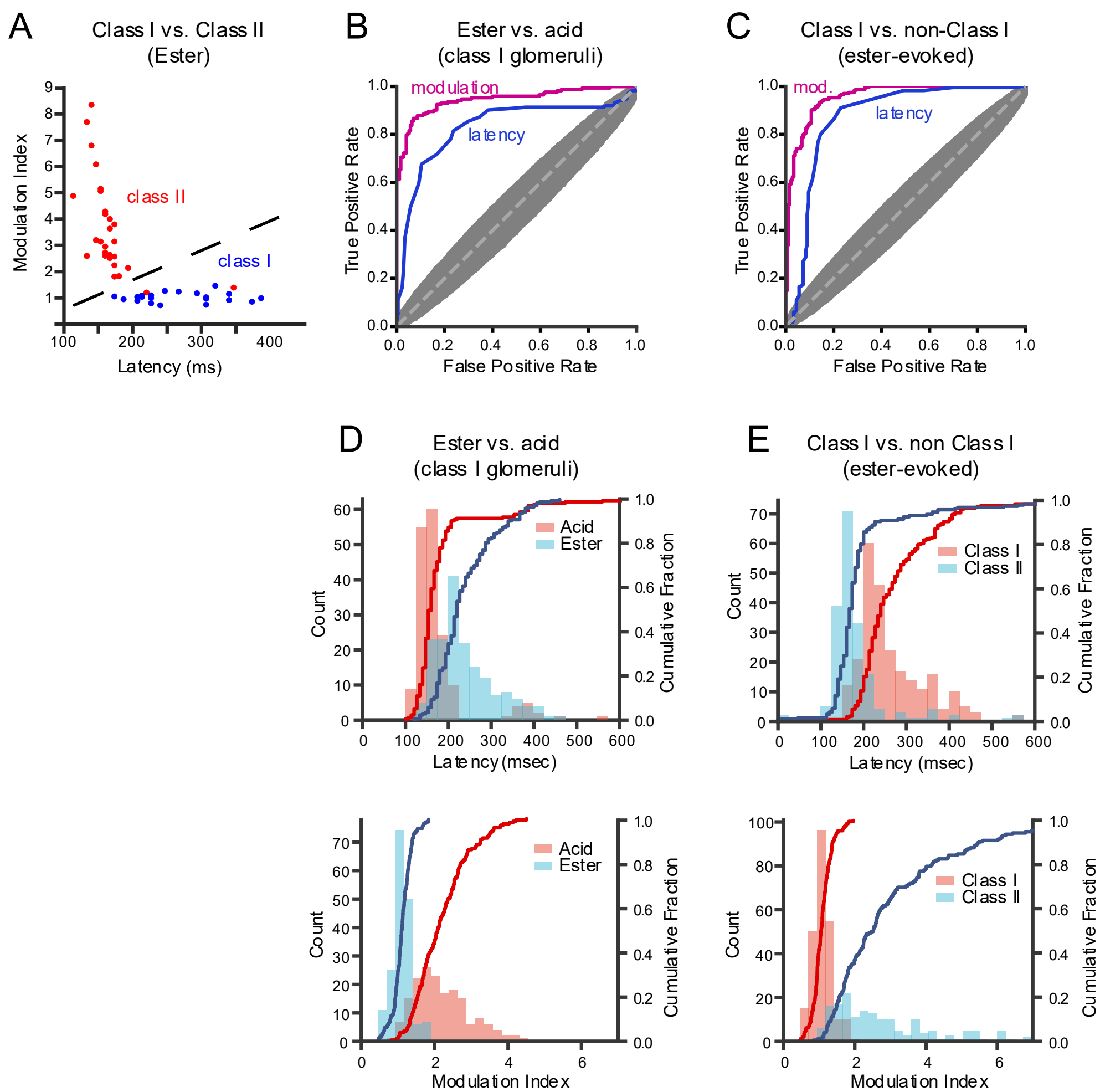

Extended Data Figure 7, related to Fig. 4. Distributions of onset latency and modulation index distinguishing acid from ester-evoked responses and class I from class II glomeruli.

A. Modulation Index versus Latency plotted for glomeruli shown in (Fig. 4C). Responses of all Ester-responsive glomeruli (methyl tiglate) grouped by Class I (blue) or Class II (red) domain location. Three responses are misclassified.

B. Receiver-operating characteristic (ROC) curves showing performance for discriminating activation of a glomerulus by a carboxylic acid or its ester derivative on the basis of onset latency or modulation index. Shaded area indicates envelope of ROC curves for scrambled data. n = 346 glomeruli from 6 OBs (4 mice).

C. Same analysis for classifying glomerular identity as class I (acid-sensitive) or class II (ester-selective) for ester-responsive glomeruli. n = 415 glomeruli from 6 OBs (4 mice).

D. Histograms and cumulative distribution function plots for onset latency (top) and modulation index (bottom) for acid- and ester-evoked responses in co-tuned glomeruli.

E. Histograms and cumulative distributions for onset latency and modulation index for ester-evoked responses in presumptive class I (acid-sensitive) and non-class I glomeruli.

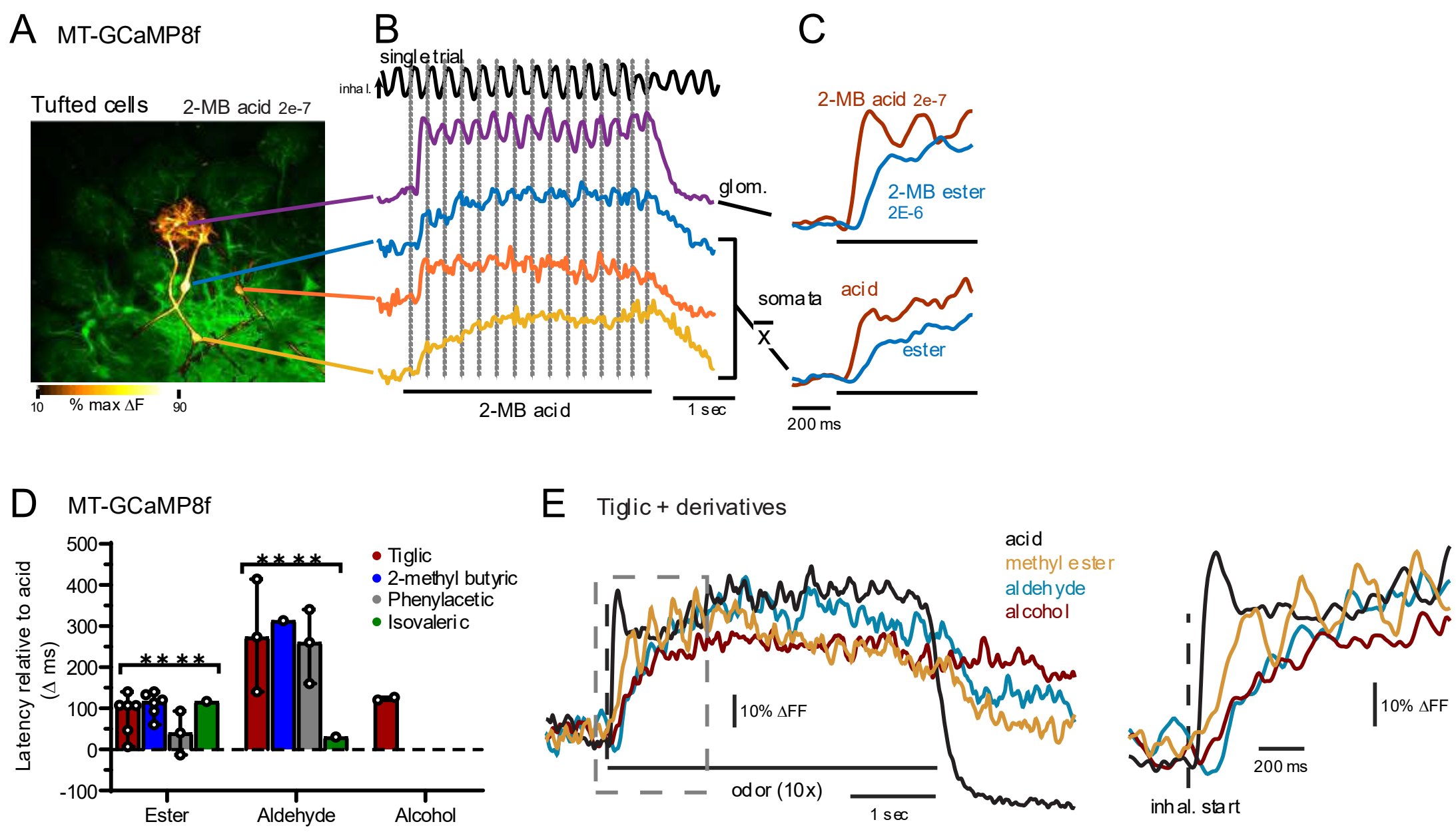

Extended Data Figure 8, Related to Figure 5. Delayed mitral and tufted cell responses to ester, aldehyde and alcohol derivatives.

A. Response map of GCaMP8f signal evoked by near-threshold 2-methyl butyric (2-MB) acid, showing singular activation of this identified glomerulus and simultaneous measurement of GCaMP8f signal in the glomerular tuft and three daughter tufted cells (TCs).

B. Time-course of signal in the glomerulus and each daughter TC, imaged in the same trial. Top trace and dashed lines show respiration measurement and inhalation onset times. Note lack of clear modulation by inhalation in somata as compared to dendritic tufts.

C. Inhalation-aligned response onsets to 2-MB acid and its methyl ester (2-MB ester) for GCaMP8f signals in the tuft (top) and averaged across the three daughter TCs (bottom), showing slower ester-evoked responses in both compartments.

D. MT-GCaMP8f glomerular onset latency delays grouped by replicates of ester, aldehyde and alcohol derivatives, relative to their parent acid, plotted for each identified glomerulus. Points indicate biological replicates per glomerulus; bars indicate median, error bars indicate range.

Esters: Δacid = 86 ± 12 ms, $t(15) = -7.4$, $p = 6E-6$, n = 16 pairs; Aldehydes: Δacid = 254 ± 36 ms, $t(7) = -7.1$, $p = 5E-5$, n = 8; Alcohols: Δacid = 120, 127 ms, n = 2). Linear mixed-effects model followed by Bonferroni-corrected pairwise comparisons of estimated marginal means (EMMs) between acids and their corresponding esters and aldehydes. Alcohols not tested (insufficient n). ****$p < 0.0001$.

E. Example traces from a tiglic acid-sensitive glomerulus showing inhalation-aligned MT-GCaMP8f response to acid, ester, aldehyde and alcohol derivatives. Right: Expansion of boxed area highlighting response onset. Odorants tested at ~10x perithreshold dilution.

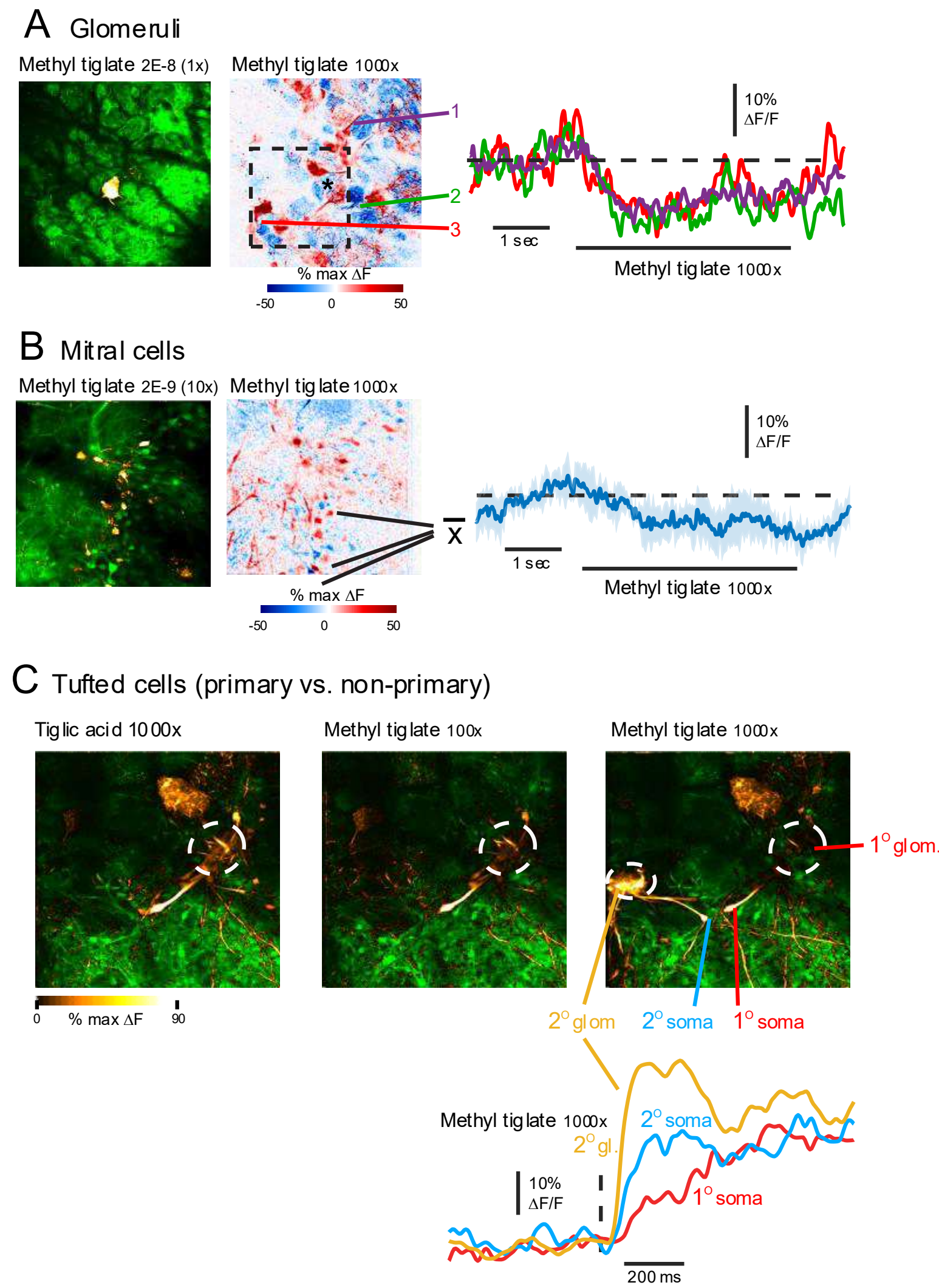

Extended Data Figure 9, Related to Figure 5. Delayed excitatory responses to ester derivatives persist despite suppression and faster excitation in neighboring glomeruli.

A. GCaMP8f responses from the glomerular layer in response to suprathreshold concentration of methyl tiglate (MT). Left-most map shows response to perithreshold MT and primary glomerulus. Right map shows response to 1000x MT, scaled and pseudocolored to show excitatory (red) and suppressive responses (blue). Asterisk indicates primary glomerulus. Note additional excited (non-primary) glomeruli as well as neighboring suppressed glomeruli. Right: Traces from three glomeruli showing suppressive responses.

B. Same as (A) for MC responses to 1000x MT. Response map overlay to 10x MT and traces from the mitral cell layer (same field of view as (C)) revealing suppressive responses in neighboring mitral cells. Trace at right shows mean signal from three MC somata.

C. Response map overlays and traces showing glomeruli and daughter TCs, imaged from same preparation, in response to suprathreshold concentrations of TA (acid) or MT (ester). Primary TA/MT glomerulus is outlined in white; note clear innervation by daughter TC. Maps sequence shows recruitment of new glomerulus with concentration increase from 100x to 1000x MT, with daughter TC soma clearly apparent (right map). Right: Traces taken from the adjacent daughter TCs innervating the primary and non-primary glomeruli (labelled 1' and 2', respectively), and the parent non-primary glomerulus ('2' gl'), in response to 1000x MT. Note that secondary glomerulus responds with shorter latency than primary, and latency differences persist in their respective daughter TCs.

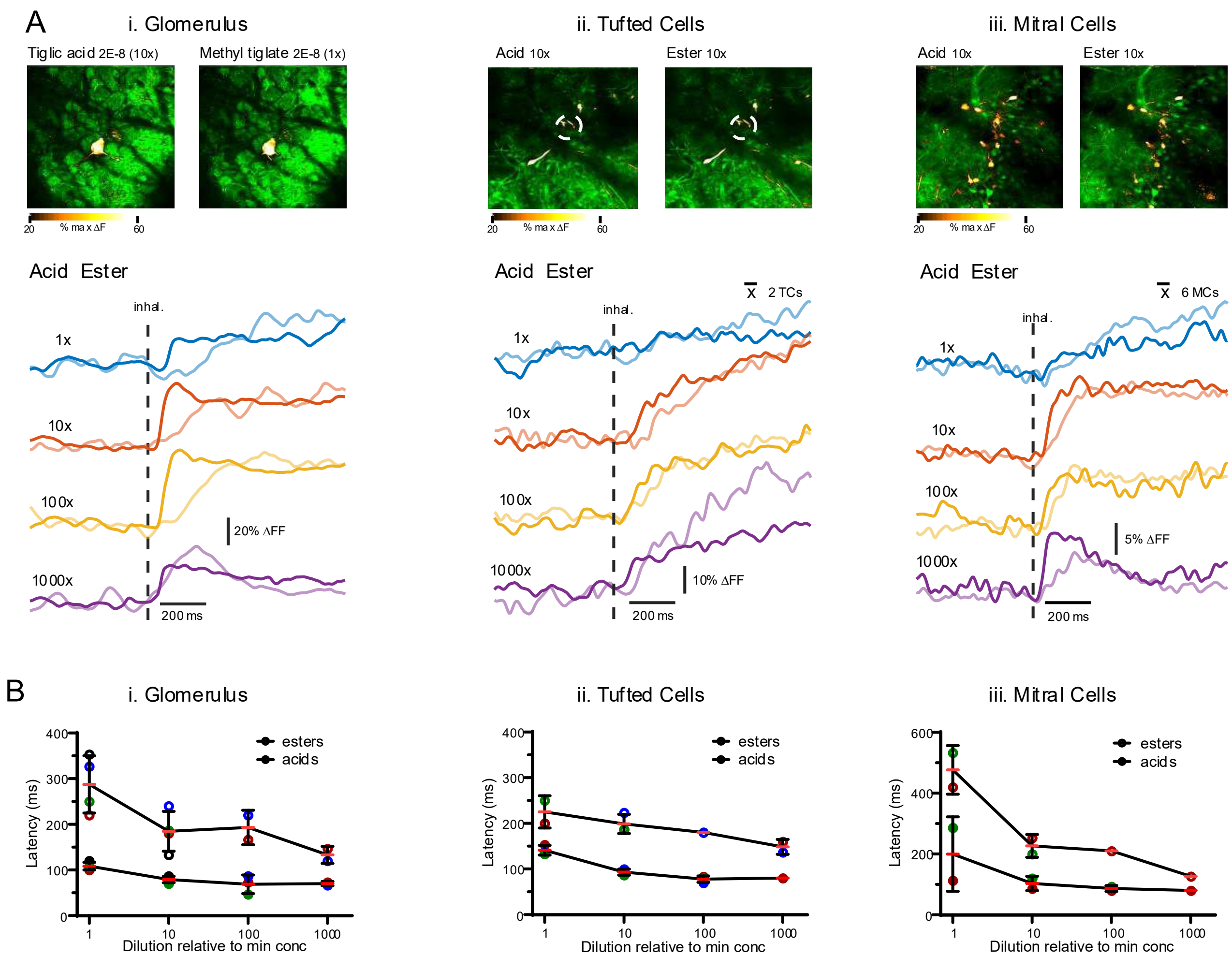

Extended Data Figure 10, Related to Figure 5. Delayed responses to ester derivatives in acid-sensitive glomeruli persist across concentration and MT cellular compartment.

A. Maps and inhalation-aligned traces of MT-GCaMP8f responses to tiglic acid or methyl tiglate in the primary glomerulus (i) or its daughter TCs (ii) or MCs (iii). Same example preparations as in Extended Data Figure S9. Top row: ΔF response overlays to perithreshold concentrations of the acid and ester, showing singular activation of the primary glomerulus (i) and identifying excited daughter cells (ii, iii). Dashed circle in (ii) indicates approximate position of parent glomerulus (our-of-plane). Below are traces to the acid and ester, matched to the same concentration relative to near-threshold (1x) are scaled as per the scale bar. All traces in a sub-panel are shown on the same absolute scale (ΔF/F). Glomerular

and mitral cell signals show reduced-amplitude response at 1000x concentration but slower excitatory response to the ester persists. Traces in (ii) and (iii) are averages across the same 2 and 6 daughter cells, respectively.

B. MT-GCaMP8f onset latencies for acid-ester pairs as a function of concentration, referenced to near-threshold dilution, for signals in primary glomeruli (i), and their daughter TCs (ii) and MCs (iii). Colored circles indicate median latency across biological replicates for a given acid or ester (same color-coding as in (A)). Red lines indicate mean of medians across acids or esters; error bars are standard deviation. All signals show longer latencies to the ester derivative at all dilutions.

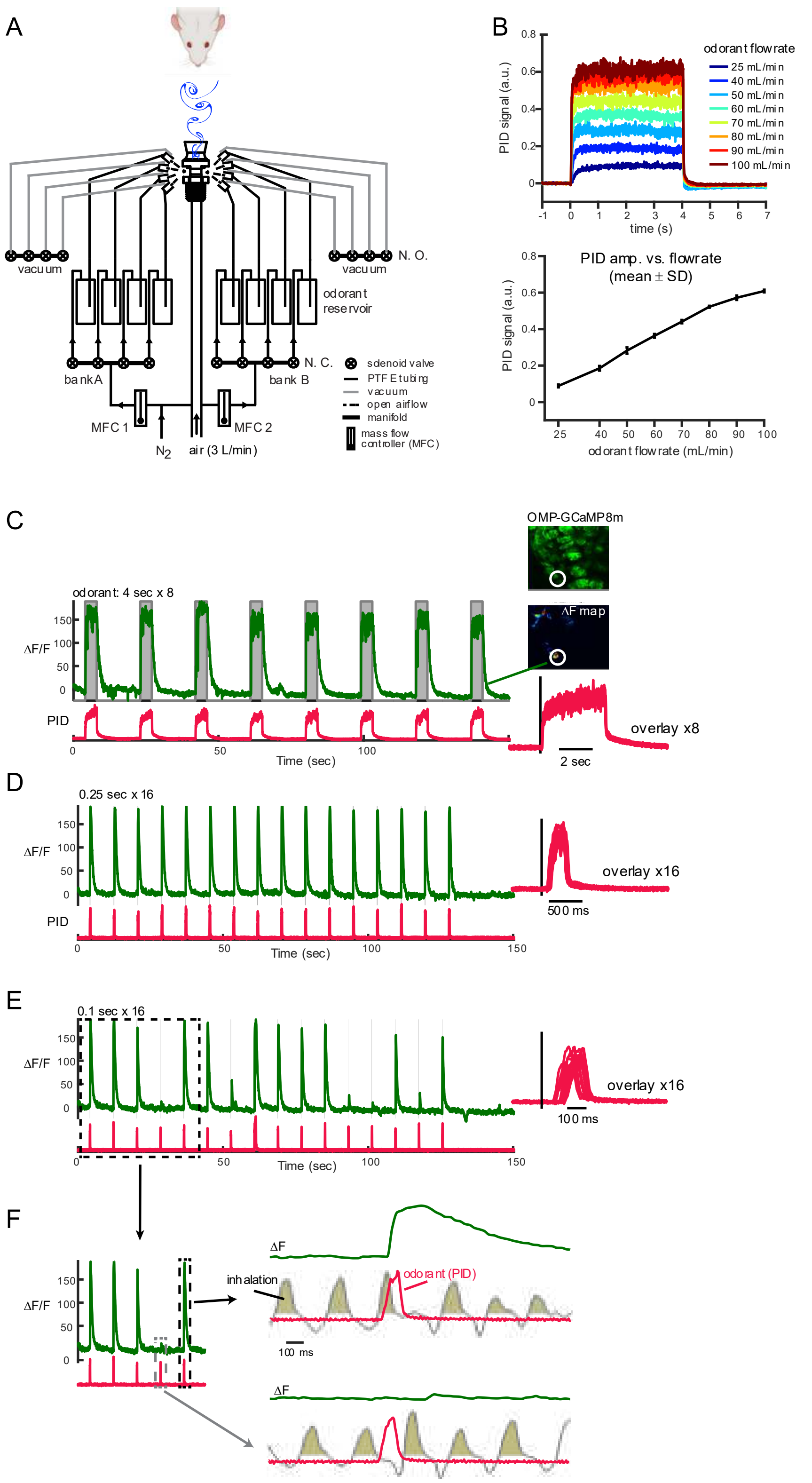

Extended Data Figure 11, Related to Methods. Design and characterization of olfactometer for control of odorant stimulus timing.

A. Schematic of olfactometer design.

B. Top: Photoionization detector (PID) traces of odorant concentration at different flowrates through odorant vial. Bottom: Mean ± SD. of PID signal amplitude versus odorant reservoir flowrate, showing a linear relationship over a four-fold range.

C. PID traces (bottom) and OSN-GCaMP8m signals evoked by repeated 4-sec odorant presentations in the awake mouse. Top insets show mean F and ΔF images with imaged glomerulus. Lower inset shows overlay of PID traces from the 8 trials.

D. Same as (C), showing consistent responses to 16 presentations of 0.25 sec duration pulse.

E. Same as (D), with 16 presentations of 0.1 sec pulse. PID trace shows some loss of precision in timing, and OSN-GCaMP8m trace shows missed responses.

F. Expansion of boxed region in (E), with respiration trace overlaid for individual presentations (right). Yellow-shaded areas indicate time period of inhalation. Note that 'missed' OSN response corresponds to odorant onset and offset occurring in between inhalations.

Odorant	CAS#	Class	Glom. ID	Ref. Dil.	Est. Conc. (M)	Figures
2-pentenoic acid	13991-37-2	acid	Or52h2	2E-6	1E-11	1
E-ethyl-pent-2-enoate	24410-84-2	ester	Or52h2	4E-6	1E-9	1
trans-2-penten-1-ol	1576-96-1	alcohol	Or52h2	2E-6	3E-10	1
2-methyl-2-pentenoic acid	3142-72-1	acid	10		1E-10	2
trans-2-hexenoic acid	13419-69-7	acid	10		1E-8	2
crotonaldehyde	123-73-9	aldehyde	10		1E-9	2
angelic acid	565-63-9	acid	10		1E-10	2
methyl angelate	5953-76-4	ester	10		1E-9	2
angelic acid isobutyl ester	7779-81-9	ester	10		1E-10	2
trans-2-pentenal	1576-87-0	aldehyde	10		1E-8	2
trans-2-hexen-1-al	6728-26-3	aldehyde	10		1E-8	2
trans-2-methyl-2-butenal	497-03-0	aldehyde	10		1E-10	2
valeraldehyde	110-62-3	aldehyde	10		1E-8	2
2-methyl-2-pentenal	623-36-9	aldehyde	10		1E-10	2
menthyl valerate	89-47-4	ester	10		1E-8	2
ethyl tiglate	5837-78-5	ester	10		1E-10	2
isopropyl tiglate	1733-25-1	ester	10		1E-8	2
hexyl tiglate	16930-96-4	ester	10		1E-10	2
benzyl tiglate	37526-88-8	ester	10		1E-10	2
propyl tiglate	61692-83-9	ester	10		1E-10	2
isobutyl tiglate	61692-84-0	ester	10		1E-10	2
geranyl tiglate	7785-33-3	ester	10		1E-10	2
isobutyl acetate	110-19-0	ester	10		1E-8	2
3-methyl-2-butenal	107-86-8	aldehyde	10		1E-8	2
2-methyl-2-butenoic acid (tiglic acid)	80-59-1	acid	10	2E-9	1E-14	2, 4, 5, 6, 7
methyl tiglate	6622-76-0	ester	10	2E-8	1E-11	2, 4, 5, 6, 7
2-methylbutyric acid	116-53-0	acid	23	2E-8	5E-13	2, 4, 5, 6, 7
methyl 2-methylbutyrate	868-57-5	ester	23	2E-8	2E-11	2, 4, 5, 6, 7
2-methylbutyraldehyde	96-17-3	aldehyde	23	2E-7	1E-10	2, 4, 5, 6, 7
2-methylbut-2-en-1-ol	4675-87-0	alcohol		2E-6	3E-10	4
2'-hydroxyacetophenone	118-93-4	ketone	12	2E-7	1E-12	5
butyric acid	107-92-6	acid		2E-4	2E-8	6
ethyl butyrate	105-54-4	ester		2E-4	1E-7	6
valeric acid	109-52-4	acid		2E-4	2E-9	2, 6
methyl valerate	624-24-8	ester		2E-4	2E-6	2, 6
2-hexanone	591-78-6	ketone	20	2E-6, 1E-4	1E-9, 1E-7	5,6
phenylacetic acid	103-82-2	acid	5	2E-6	5E-13	4, 5, 7
ethyl phenylacetate	101-97-3	ester	5	2E-4	1E-9	4, 5, 7
isovaleric acid	503-74-2	acid	11	2E-8	5E-13	2, 4, 5, 7
isovaleraldehyde	590-86-3	aldehyde	11	2E-6	5E-9	4, 5, 7

Extended Data Table 1. Odorants, reference dilutions and estimated concentrations used.
'Glom ID', identifier for primary glomerulus associated with each odorant; numbers refer to identifier of functionally-identified glomerulus in ref. 9.
